# Sildenafil 4.0—Integrated Synthetic Chemistry, Formulation and Analytical Strategies Effecting Immense Therapeutic and Societal Impact in the Fourth Industrial Era

**DOI:** 10.3390/ph14040365

**Published:** 2021-04-15

**Authors:** Andreas Ouranidis, Anastasia Tsiaxerli, Elisavet Vardaka, Catherine K. Markopoulou, Constantinos K. Zacharis, Ioannis Nicolaou, Dimitris Hatzichristou, Anna-Bettina Haidich, Nikolaos Kostomitsopoulos, Kyriakos Kachrimanis

**Affiliations:** 1 Department of Pharmaceutical Technology, School of Pharmacy, Aristotle University of Thessaloniki, 54124 Thessaloniki, Greece; anastsia@pharm.auth.gr (A.T.); evardaka@pharm.auth.gr (E.V.); 2 Department of Chemical Engineering, Aristotle University of Thessaloniki, 54124 Thessaloniki, Greece; 3 Laboratory of Pharmaceutical Analysis, Department of Pharmaceutical Technology, School of Pharmacy, Aristotle University of Thessaloniki, 54124 Thessaloniki, Greece; amarkopo@pharm.auth.gr (C.K.M.); czacharis@pharm.auth.gr (C.K.Z.); 4 Laboratory of Pharmaceutical Chemistry, Department of Pharmaceutical Chemistry, School of Pharmacy, Aristotle University of Thessaloniki, 54124 Thessaloniki, Greece; inikolao@pharm.auth.gr; 5 Department of Urology, Medical School, Aristotle University of Thessaloniki, 54635 Thessaloniki, Greece; djchatzi@auth.gr; 6 Department of Hygiene, Social-Preventive Medicine and Medical Statistics, Medical School, Aristotle University of Thessaloniki, 54124 Thessaloniki, Greece; haidich@auth.gr; 7 Center of Clinical, Experimental Surgery and Translational Research, Biomedical Research Foundation of the Academy of Athens, 11527 Athens, Greece; nkostom@bioacademy.gr

**Keywords:** sildenafil, fourth industrial era, efficacy and safety, improved solubility, nanomaterial, formulation, PDE5 inhibitors, reduced side effects

## Abstract

Sildenafil is a potent selective, reversible inhibitor of phosphodiesterase type 5 (PDE5) approved for the treatment of erectile dysfunction and pulmonary arterial hypertension. Whilst twenty years have passed since its original approval by the US Food and Drug Administration (USFDA), sildenafil enters the fourth industrial era catalyzing the treatment advances against erectile dysfunction and pulmonary hypertension. The plethora of detailed clinical data accumulated and the two sildenafil analogues marketed, namely tadalafil and vardenafil, signify the relevant therapeutic and commercial achievements. The pharmacokinetic and pharmacodynamic behavior of the drug appears complex, interdependent and of critical importance whereas the treatment of special population cohorts is considered. The diversity of the available formulation strategies and their compatible administration routes, extend from tablets to bolus suspensions and from per os to intravenous, respectively, inheriting the associated strengths and weaknesses. In this comprehensive review, we attempt to elucidate the multi-disciplinary elements spanning the knowledge fields of chemical synthesis, physicochemical properties, pharmacology, clinical applications, biopharmaceutical profile, formulation approaches for different routes of administration and analytical strategies, currently employed to guide the development of sildenafil-based compositions.

## 1. Introduction

From a serendipitous discovery involving an experimental hypertension and angina medication to the first oral treatment indication of erectile dysfunction, the development of sildenafil (widely recognized by the name of its commercial blockbuster formulation Viagra^®^), is unique. Sildenafil is a potent selective, reversible inhibitor of phosphodiesterase type 5 (PDE5) also repurposed for the treatment of pulmonary arterial hypertension (PAH) under the Pfizer brand name Revatio^®^ [1]. Moreover, due to the high levels of PDE5 expression in the lung tissue and the proven vasodilatory activity of sildenafil, researchers rationally considered the drug’s possible therapeutic effect against pulmonary fibrosis, a complication of the COVID-19 disease [2,3,4].

Sildenafil belongs to the class II of the Biopharmaceutical Classification System (BCS) and its solubility is highly dependent on pH levels. The citrate sildenafil salt initially dehydrates and then thermally decomposes at 189.4 °C, accompanied by the simultaneous melting of the derivative product. Since sildenafil shares a common metabolic pathway with numerous drugs, interactions have been documented during co-administration. The major metabolite derived by *N*-demethylation, is *N*-desmethylsildenafil (UK-103,320) and exhibits approximately 50% in vitro activity in PDE5 inhibition, thus constituting the 40% of the overall fraction of the observed systemic exposure. *N*-desmethylsildenafil is further metabolized demonstrating a terminal half-life of 4 h [5,6,7].

Oral formulations prevalent in clinical practice, are the standard dosage form of choice taxonomized as tablets, chewables, capsules, suspensions and liquids. Tablets provide technological formulation advantages and reasonable compliance, although alternative administration routes have also been proposed in merit. Under this lens, researchers have developed a variety of bioequivalent formulations absorbed by sublingual, supralingual, intranasal, inhalable, transdermal and intravenous administration routes.

Several analytical protocols have been recruited for the accurate determination and quantification of sildenafil in pharmaceutical, dietary products, herbal products and biological matrixes. In this comprehensive review study, we will attempt to investigate the chemical synthesis, physicochemical properties, pharmacology, clinical applications, biopharmaceutical profile, formulation approach for several administration routes and analytical strategies, employed to guide the development of novel sildenafil-based compositions.

## 2. Chemical Synthesis of Sildenafil

**Figure 1:**
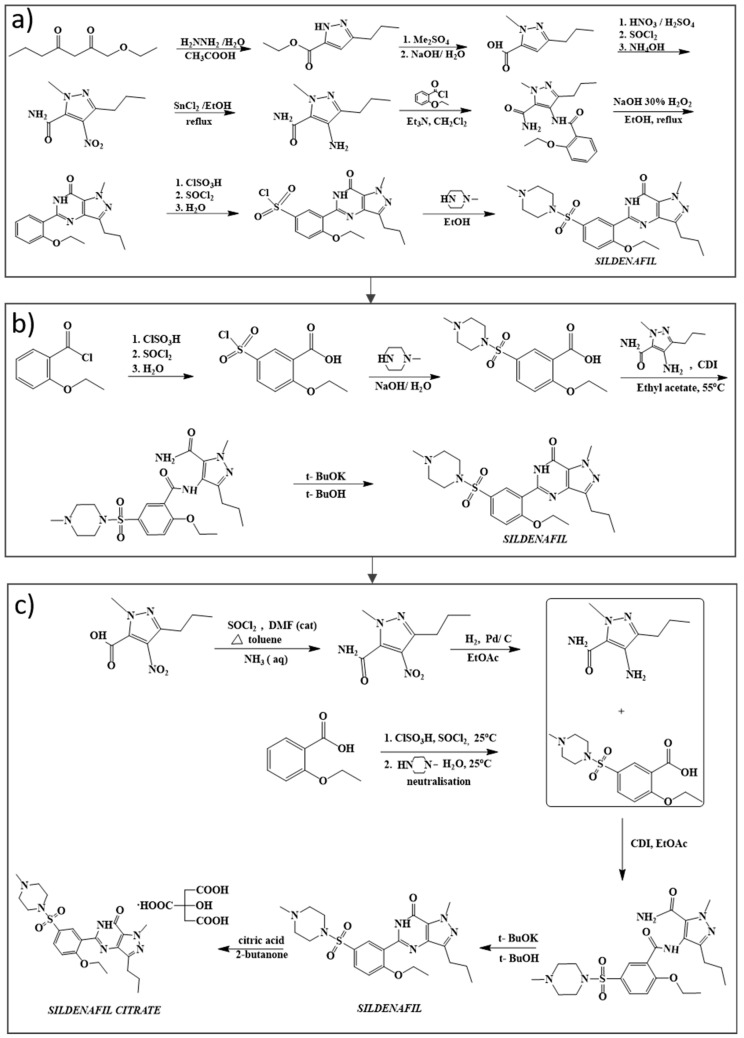
Evolution of sildenafil synthetic routes: (**a**) initial synthesis of sildenafil citrate; (**b**) improved synthesis scheme using 2,2-ethoxy benzoyl chloride; (**c**) optimized commercial synthesis of sildenafil in terms of green chemistry.

The chemical name of sildenafil is 5-[2-ethoxy-5-(4-methylpiperazin-1-ylsulfonyl) phenyl]-1-methyl-3-n-propyl-1,6-dihydro-7*H*-pyrazolo[4,3-d]pyrimidin-7-one. Sildenafil (Figure 1a–c lower right) was the first API structure rationally developed utilizing computational drug-design protocols targeting the inhibition of phosphodiesterase type 5 isoenzyme [8].

The first synthetic route of sildenafil (Figure 1a) accomplished the preparation of its pyrazole derivative from ethyl 3-butyrylpyruvate and hydrazine hydrate in acetic acid, followed by the selective N-methylation of the pyrazole ring with dimethyl sulfate. The carboxylic acid was obtained after alkaline hydrolysis was subjected to nitration, followed by treatment with concentrated ammonium hydroxide solution to sequentially deliver the corresponding carboxamide derivative. The nitro group of the mentioned carboxamide derivative was then reduced to an amino group by stannous chloride/hydrochloric acid in ethanol, leading to the formation of the main 4-aminopyrazole structure. Mild amidation of the aminopyrazole derivative by the appropriate benzoyl chloride was performed, followed by cyclization mediated by hydrogen peroxide under basic environment which led to the formation of pyrimidinone heterocycle ring. Chloro-suIphonylation of pyrimidinone derivative imposed selectively on the 5′ position of the phenyl ring, led to the aroyl sulfonyl chloride derivative which was then coupled with N-methylpiperazine to afford sildenafil (Figure 1a) [8].

The first-generation synthetic process was not deemed suitable for scale-up due to safety and environmental issues and therefore alternatives were successfully developed. In the improved synthesis scheme presented by Figure 1b, 2-ethoxybenzoyl chloride was exploited as the starting material and reacted with chlorosulphonic acid in the presence of thionyl chloride, in order to transform the intermediate sulphonic acid to the corresponding sulphonyl chloride. The arylsulfonyl chloride derivative was hydrolyzed in a neutral environment to form 5-(chlorosulfonyl)-2-ethoxybenzoic acid, which was coupled with N-methylpiperazine in the presence of aqueous sodium hydroxide, producing the corresponding sulfonamide derivative. Then the sulfonamide product was reacted with the corresponding aminopyrazole derivative in the presence of *N*,*N*′-carbonyldiimidazole. The pyrimidone heterocycle ring of sildenafil is formed by adding potassium *t*-butoxide in *t*-butanol in order to minimize the formation of side products and close to quantitative yield [9,10].

The commercial synthesis of sildenafil was further developed in the context of 4.0 green chemistry directives, aiming to advance the environmental performance during large scale production (Figure 1c). The removal of the tin chloride reduction, the use of stoichiometric quantities of thionyl chloride with a solvent in the preparation of sulphonyl chloride intermediate, the elimination of hydrogen peroxide from the synthesis and the use of thionyl chloride rather than oxalyl chloride for the formation of 2-ethoxybenzoyl chloride in effort to avoid carbon monoxide emissions, were the main of alterations applied to the early batches. The targets of this redesigned synthesis informed the achievement of a clean cyclization reaction as the final step. Expanding on this, both the use of water instead of organic solvents for the preparation of the sulfonamide and of toluene as a solvent with minimized thionyl-chloride equivalents, improved the environmental burden of the earlier steps. Under this lens toluene was actually deemed fit for recovery and reuse, significantly lowering the cost of the overall process. In addition, the use of N,N-carbonyldiimidazole (CDI) in ethyl acetate as solvent proved a very simple process in order to give the desired amide which was crystallized directly from the reaction mixture. Due to the latter, the CO_2_ emissions correlated to the reaction of either thionyl chloride or oxalyl chloride with ethyl acetate were further diminished and the obtained yield of sildenafil citrate was 75% while potential toxic materials were sidestepped, leading to a high quality and environmentally clean product [11].

## 3. Physiochemical Properties

Sildenafil citrate and sildenafil base are white to off-white crystalline solids presenting densities of 1.59 and 1.17 g/cm^3^, respectively [12]. The sildenafil free base form precipitates as a fine crystalline powder in 0.02M solution of sildenafil at pH 8, achieved by adding a 0.05 M solution of NaOH dropwise [12]. According to the BCS nomenclature and the regulatory biowaiver guidelines, sildenafil citrate is classified as an active pharmaceutical ingredient (API) of low solubility and high membrane permeability, even though it meets the criteria of highly soluble API at pH 4 environments [13]. Enhancement of amorphous sildenafil’s physical stability has been evaluated by investigating the impact of Tg polymer additives [14].

Moreover, sildenafil faces polymorphic transition and has been produced as salt, co-crystal or solvate Table 1. The most common crystal forms of sildenafil are the monoclinic Form I and the orthorhombic Form II [15], while the most common salts are sildenafil citrate monohydrate and sildenafil saccharinate [16].

### 3.1. Solubility

**Figure 2:**
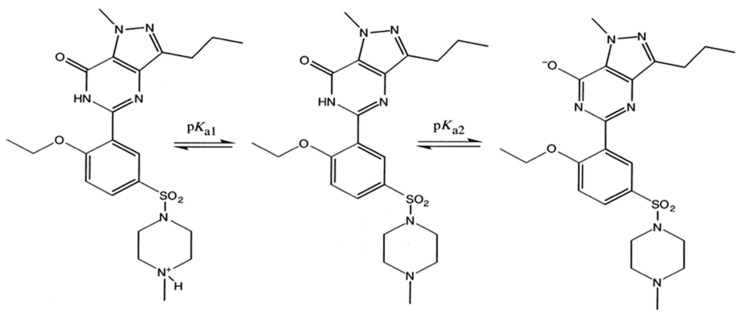
Ampholytic ionization states of sildenafil (Reprinted from ref. [14]).

Pure sildenafil (base) is soluble in diethylene glycol monoethyl ether (Transcutol^®^) 24.7 ± 4.7 mg/mL and in ethanol (5.2 ± 1.2 mg/mL) but insoluble in distilled water. Pharmaceutical manufacturers managed to improve the molecule’s water solubility by citrate salt formation. Sildenafil citrate is soluble in distilled water 4.1 ± 1.3 mg/mL, in dimethyl isosorbide 9.98 ± 0.79 and in various oils like Miglyol 4.23 ± 0.39 mg/mL and oleic acid 6.77 ± 0.54 mg/mL [18,26]. An API’s solubility is intrinsically dependent on the variation of pH levels. At a temperature of 37 °C in an aqueous environment at neutral or slightly acidic pH~4 conditions, the API demonstrates the lowest and the highest of solubility boundaries, namely 0.02 mg/mL and 7.1 mg/mL, respectively [27]. This dual ampholytic behavior is attributed to the endogenous molecular nature of the sildenafil chemical structure which encompasses structural moieties such as the moderately strong, basic functional NH-piperazine featuring a pK_a1_ value of 6.78 and the weak acidic HN- pyrimidone amide featuring pK_a2_ value of 9.12, (Figure 2) [28,29].

### 3.2. Permeability

Sildenafil citrate as an ordinary ampholyte, i.e., combining moderate basicity and weak acidity character as shown by Figure 2, exhibits permeability coefficient changes dependent on the given pH values. As a weak basic compound, it becomes only partially ionized at neutral pH environments while the higher permeation rates are correlated to pH values of 8–11 [29,30]. The strongest permeability coefficient is determined at pH 7.4, whereas absolute ionization levels are estimated approximately at 43%, indicating that the combined contribution of both the ionized and neutral species facilitate the permeation event [31].

### 3.3. Thermogravimetric Analysis (TGA)

Thermogravimetric analysis (TGA) methods have been employed for the reliable, qualitative assay of sildenafil citrate products. Sildenafil citrate undergoes thermal decomposition at 189.4 °C accompanied by the almost simultaneous melting of the decomposition product. Moreover, the said temperature overlaps with the decomposition of the pure citric acid component, specifically at 189.7 °C [12]. In the temperature range between 190 °C and 229.5 °C, sildenafil citrate loses mass between 22–28.8% *w*/*w*, a phenomenon corresponding to the citric acid evaporation [32,33]. The remaining mass after heating to 230 °C is pure sildenafil base while at elevated temperature conditions i.e., over 400 °C a low, yet continuous mass decrease is related to elemental carbon formation [34].

### 3.4. Differential Scanning Calorimetry (DSC)

The DSC curve of sildenafil citrate exhibits sharp endothermic events at 196–204 °C depending on the scanning conditions, while no sign of decomposition is noted up to 229.5 °C [32,33,34]. Sildenafil anhydrous form I produces a rather simple thermogram, presenting a sharp endotherm peak at 188–189 °C. Regarding sildenafil form II, it presents two overlapping events endothermic/exothermic before melting at the same temperature, indicating the transformation to form I through a two-steps process, involving a glass-like solid [15].

### 3.5. Infrared Spectroscopy (IR)

Sildenafil citrate and sildenafil base produce similar spectra except from the broad bands, i.e., lower than 3411 cm^−1^, which represent the O-H stretching of the citrate [35]. The most characteristic peak of both substances is due to the -C=O vibration, appearing in a band of strong intensity at 1703 cm^−1^. Other characteristic bands are considered: the stretching of the secondary amides N-H at 3300 cm^−1^, the N-H bending vibration at 1650-1580 cm^−1^, the asymmetrical stretch of S=O at 1359 cm^−1^ and the symmetrical one at 1172 cm^−1^ [35]. IR measurements have been widely used as a fast qualitative method for the analysis of adulteration of natural products (marketed herbal aphrodisiacs) with sildenafil [36,37] and also for the detection of counterfeit tablets [38,39].

### 3.6. X-ray Crystallography Data

Several methods have been tested to advance the crystallization process of sildenafil i.e., solution crystallization, antisolvent addition, reflux and slow solvent evaporation [35]. The molecule is built around a rigid central core fragment which consists of a π-conjugated bicyclic pyrazolopyrimidone and a phenyl ring. The bulky methylpiperazine-sulfonyl fragment appears to be insensitive to its rotation in respect to the central core. The flexible part of the molecule is attributed to the propyl group adopting three positions accounted for the equal known derivatives [16]. Moreover, as confirmed by the X-ray patterns the base molecule when compared to the salts, exhibits poor crystallinity (forming rod-shaped crystals), presenting refractive indices of 1.38 and 1.53, respectively [12]. The crystal structure of the free base is monoclinic system, bearing the space group P21/c with unit cell parameters: [a = 17.273 (1), b = 17.0710 (8), c = 8.3171 (4)] Å, angle β = 99.326 (2), Z = 4, V = 2420.0 (3) Å3 [16]. Conversely, the crystal of sildenafil citrate adopts the orthorhombic system with the space group Pbca and unit cell parameters of a = 24.002 (4), b = 10.9833 (17), c = 24.363 (3) Å, Z = 8, V = 6422.9 (17) Å3 [40]. The crystal structure of the anhydrous sildenafil citrate remains unsolved.

### 3.7. NMR Spectroscopy

A comprehensive comparative study between sildenafil citrate and sildenafil base has been published by Wawer et al. who examined the differences of sildenafil’s spectra in solution, in solid state and in pharmaceutical dosage forms. Specifically, in the ^1^H-NMR spectra they underline that the difference between the base and salt is significant regarding the piperazine fragment where the methylated N is the most probable place of protonation [41]. In addition, the ^13^C-NMR presents various chemical shifts which differ between the different states of sildenafil, thus revealing the rigidity of the compound which can be explained by intermolecular hydrogen bonds by ^15^N-NMR [41]. The ^13^C-MAS NMR technique has been also exploited by researchers to reveal the location of the water molecules and their mobility in sildenafil citrate crystal structure, combined with molecular dynamic simulations and XRD measurements [42].

## 4. Pharmacology and Clinical Applications

### 4.1. Treatment of Erectile Dysfunction

Sildenafil citrate acts as a potent selective, reversible inhibitor of phosphodiesterase type 5 (PDE5), i.e., the enzyme responsible for the hydrolysis of cyclic guanosine monophosphate (cGMP), found in excess in the penis corpus carvenosum, as well as in the smooth muscle cells of vessel walls and the lung tissue [1]. During sexual stimulation, nitric oxide released by the penile terminal nerves and endothelium activates the soluble guanylate cyclase, which in turn mediates the conversion of guanosine triphosphate (GPD) to cyclic guanosine monophosphate (cGMP). Therefore, increase of cGMP induces smooth muscle fiber relaxation by minimizing the intracellular calcium concentration. The potency and duration of action of cyclic nucleotides is controlled by phosphodiesterases type 5 which degrade the cGMP. Conclusively, the inhibition of PDE5 by sildenafil upon local release of NO caused by sexual arousal signaling, fosters the high-level cGMP availability in the corpus carvenosum area, underpinning the vasodilation response, i.e., contributing to the consequent increased local blood flow levels responsible for the erectile response [23,24].

### 4.2. Treatment against Pulmonary Arterial Hypertension

Sildenafil (as Revatio^®^) is approved for the treatment of pulmonary arterial hypertension (PAH) for the recommended dose regime of 20 mg, three times a day. PAH refers to a rare blood vessel disorder occurring to the major pulmonary artery, responsible for transporting blood volumes from the right ventricle of the heart to the lungs. The disease is defined by the three scenarios, namely the sustained raise of pulmonary artery (i) systolic pressure > 30 mmHg during exercise, (ii) mean pressure > 25 mmHg at relaxation and (iii) wedge pressure < 15 mmHg [43,44,45]. Since PDE5 enzymes are prevalently expressed also in the smooth muscle cells of the pulmonary vasculature, the use of sildenafil as inhibitor of the iterated isoenzymic system promotes the activity of nitric oxide-cyclic guanosine monophosphate pathway, thus leading to relaxation of the arterial walls of the pulmonary arteries [46]. For PAH patients this action translates in the controlled decrease of pulmonary blood pressure and to a slighter extend, in a desired systemic vasodilatation effect [47].

### 4.3. Sildenafil as a Potential Treatment Option for COVID-19

Due to the high levels of PDE5 expression in the lung tissue and the proven vasodilatory activity of sildenafil, researchers rationally considered the drug’s possible repurposing against pulmonary fibrosis, i.e., a harsh complication of the COVID-19 disease. Elaborating on this, sildenafil is proposed to inhibit intrapulmonary vasoconstriction induced by the Ang II type I (AT1) receptor regulation due to SARS-CoV-2-ACE2 binding to alveolar cells, bronchial epithelium and vascular endothelium. The latter is achieved by targeting the NO/cGMP/PDE5 pathway, since NO acts as an inhibitor of the AT1 receptor. Also, it has been proposed that sildenafil through the inhibition of PDE5 receptors mediates the regulation of several pathways of smooth muscle cells of the pulmonary vasculature, preventing the spread of local inflammation by imposing the reduction of the release of proinflammatory cytokines.

Additionally, according to another emerging scientific hypothesis, sildenafil might potentially contribute a protective effect on the pulmonary microangioma which is also affected by SARS-COV-2 [48]. Qiao et al. after performing in silico studies, reported the high binding affinity of sildenafil with SARS-CoV-2 3CL protease (docking score -8,9 kcal/mol), suggesting a promising inhibition towards the said enzyme, one that favors the conformational changes of the SARS-Cov-2 virus [49]. Clinical trials involving the use of sildenafil on patients with COVID-19 are ongoing. The first open label, pilot efficacy and safety study (NCT04304313) incorporated 10 participants while the second randomized (NCT04489446) placebo-control trial recruited 40 patients suffering from perfusion anomalies, another recently described complication of COVID-19. Even though there exist evidence that sildenafil could be applied in therapeutic protocols against SARS-Cov-2 virus, a safe conclusion has yet to be reached based on further research and on a larger clinical scale.

### 4.4. Potential Sildenafil Effectiveness on Cancer Treatment and Type 2 Diabetes

Lately, the anticancer efficacy of sildenafil has been reported by several researchers. In a recent study, the inhibition of PDE5 by high-affinity inhibitors was suggested as a chemoprotective approach for colorectal cancer. Oral administration of sildenafil in mice with inflammation-induced colorectal cancer led to significant suppression of cancer cells proliferation, especially in early stages of carcinogenesis, compared to mice not receiving sildenafil [50]. Antitumor effect of sildenafil in colorectal cancer has been also reported for human cell lines in vitro and in vivo. Sildenafil remarkably inhibited cell multiplicity by causing cell cycle arrest and apoptosis with augmented intracellular reactive oxidative species (ROS) levels [51]. Additionally, combination of curcumin with sildenafil improved the effectiveness of 5-flurouracil and anti-PD-1 antibody against CT26 colorectal tumor, offering novel therapeutic potential in already established chemotherapy treatments [52]. In another research, sildenafil inhibited cancer cell growth in vitro by causing caspase-dependent apoptosis of B-chronic lymphocytic leukemia cells [53]. Furthermore, combination of sildenafil with doxorubicin was proposed as a neoadjuvant treatment for prostate cancer. A sildenafil and doxorubicin combination induced significant suppression of tumor development and upgraded caspase-3 activity as well as apoptosis, while sildenafil acted as cardioprotective agent against doxorubicin-induced cardiotoxicity effect [54]. Similar results were reported after co-treatment of those agents in breast cancer cells [55]. Sildenafil was also found to augment the efficacy of other chemotherapeutic drugs in bladder and pancreatic tumor cells including mitomycin C, doxorubicin, cisplatin, and gemcitabine [56].

Interesting findings have been also reported concerning the administration of sildenafil in patients with type 2 diabetes. Specifically, administration of 50 mg of sildenafil citrate for a month to male patients with type 2 diabetes reduced microalbuminuria and the percentage of glycosylated hemoglobin in the serum, improving endothelial function and metabolic control [57]. Similarly, improvement of endothelial function and lessening in vascular inflammation markers was observed after regular administration of sildenafil in diabetic patients [58]. In a three-month clinical trial conducted on male patients suffering from diabetes mellitus, administration of 100mg of sildenafil per day led to enhanced glucometabolic control, suggesting further research in the role of PDE5 inhibitors in type 2 diabetes [59].

## 5. Pharmacokinetic and Pharmacodynamic Profile

### 5.1. Pharmacokinetic Profile of Sildenafil

**Figure 3:**
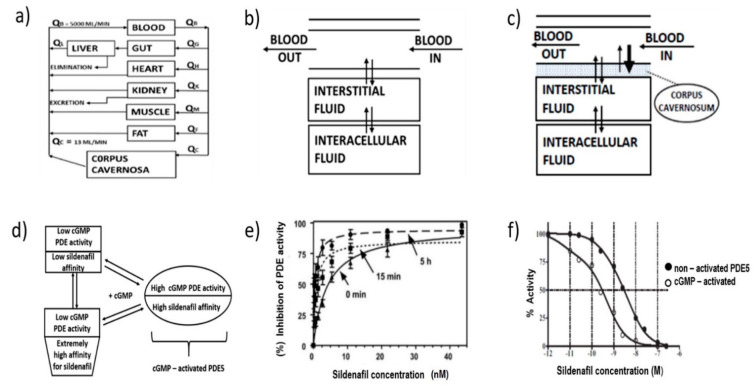
Pharmacodynamic profile of sildenafil: (**a**) distribution flows in a physiological map of human tissues; (**b**) schematic of artery and interstitial/intracellular medium, intracellular tissue; (**c**) schematic of artery and interstitial/intracellular medium, intracellular tissue pinpointing the corpus cavernosum compartment; (**d**) conversion of the catalytic center of the PDE5 enzyme into a higher affinity structure; (**e**) enhancement of inhibition of PDE activity by increase of sildenafil concentration (adopted with permission from [70]. 2007 ASPET); (**f**) differential affinities of sildenafil inhibition activated by cGMP (adopted with permission from [72]. 2010 ASPET).

Per os administration of sildenafil tablets relates to the frequent dose regime route and formulation strategy, respectively. Sildenafil is highly absorbed and follows the distribution scheme presented in overview by Figure 3a and in detail by Figure 3b,c; the resulting absolute bioavailability does not exceed 41%, due to the extensive first-pass metabolism effect. Maximum plasma concentration, i.e., C_max_ in fasted condition is achieved within 30 to 120 min, while co administration with food reduces the absorption rate, delaying the T_max_ by 60 min while decreasing C_max_ by 29% [60]. Several studies reported that sildenafil is primarily degraded via the cytochrome P450 enzymes CYP3A4 (major route) and CYP2C9 (secondary route), while only limited biotransformation of the drug is attributed to the isozymes CYP2D6 and CYP2C19.

The major metabolite of sildenafil, N-desmethylsildenafil (UK-103,320) is derived from N-demethylation and exhibits approximately 50% in vitro activity in PDE5 inhibition, in relation to the ancestor compound. Plasma concentration of this metabolite constitutes approximately 40% of the fraction of the observed systemic exposure for sildenafil and is further metabolized, demonstrating a terminal half-life of approximately 4 h [5,6,7]. Sildenafil is efficiently distributed to the tissues, as proclaimed by its high volume of distribution (105 L). Both the parent drug and the major metabolite show high albumin binding rates of 96%, yet this binding affinity does not appear to affect the overall plasma concentration [60]. The clearance capacity of sildenafil has also been estimated to 41 L/h and the main elimination root after intravenous or oral administration for all metabolites is through feces (80–88%), while in a limited extend excretion occurs through urines (about 13%) [61].

### 5.2. Pharmacodynamic Properties

The selectivity of sildenafil towards PDE5 inhibition is well established. The half-maximal inhibition (IC_50_) of PDE5 has been estimated at 3.5 nmol/L, approximately 10-fold greater than IC_50_ rate for inhibition towards PDE6, i.e., the enzyme responsible for the phototransduction pathway of the retina. In addition, sildenafil was found 80 times less potent as inhibitor for the PDE1 receptors and more than 700 times less potent for other isozymes of the phosphodiesterase family. Expanding on this, the selectivity for PDE5 was measured 4000-fold higher than cAMP-specific PDE3, which acts as mediator for the mechanism of cardiac contractility [62].

Sildenafil is a mild transient vasodilator similar to the less potent of nitrates, with the ability to maintain hemodynamic balance in both healthy and ischemic heart disease patients. Post administration, a slight decrease in systemic vascular resistance is observed due to augmentation of the endogenous NO-cGMP metabolic pathway. As a consequence of this vasodilatory effect, a moderate reduction of both systolic and diastolic blood pressure has been recorded, after administration of 100 mg sildenafil—without presenting significant escort changes of the heart rate [63]. Moreover, the main adverse events of headache and flushing are attributed to vasodilation [64]. Mild visual disturbances and in particular color perception disorders, associated to PDE6 inhibition are also observed [65].

Sildenafil single dose per os has been found to be effective in 60% and 33% of males at 8 h and 12 h [66] post administration. Particularly, high pharmacological response rates (74%) in sildenafil treatment have been reported at 12 h after the administration, in open titrated response studies [67]. The effects appear to last for more than 24 h in patients who have recently begun treatment [68]. Sildenafil is accumulated and maintained in vascular smooth muscle cells of the penis due to its high affinity to PDE5 (Figure 3c) [69]. In Figure 3d, the dynamic presence of the two known conditions for the inhibition of cGMP-activated PDE5 by sildenafil, is presented. Upon cGMP increase, sildenafil converts the catalytic center of the PDE5 enzyme into a higher affinity structure that strongly prevents the hydrolysis of cGMP (Figure 3e,f) [70]. Due to the conformational swing of the enzymic active site, sildenafil concentration levels of 1nM appear fit to induce in vitro 80% of PDE absolute inhibition (Figure 3f). Elaborating on this PDE5 (6 μM) was incubated for 15 min, or for 5 h at 4 °C with or without the cGMP (2.5 mM). After dissolution, the enzyme was added to the reaction test mixture containing increasing concentrations of sildenafil (above curve).

As presented by Figure 3f, incubation in the presence of cGMP increases the inhibition of the catalytic activity of PDE. This discovery has been validated by clinical information demonstrating that the drug remains potent in chronic periods beyond the nominal half-life [67,71]. PDE5 displays two high homology regulatory regions i.e., GAF-A and GAF-B. Several research groups [67,72] also demonstrated that PDE5 is converted from a low- to a high-activity state upon cGMP binding to GAF-A region (EMBO J 22: 469–478, 2003). Upon this incidence the apparent affinity of sildenafil is consequently upgraded from the range of nanograms to that of picograms, providing irrefutable evidence for the emergence of a sensitivity state towards PDE5 inhibition. The presence of the dual conformational conditions was clinically supported by [70,73].

### 5.3. Drug Interactions

Since inhibition of PDE5 enhances the hypotensive effect of NO, the administration of sildenafil to patients receiving organic nitrates is contraindicated [74]. However, in a recent study, the combination of single 50 mg doses of sildenafil with 10 mg single doses of isosorbide mononitrate (ISMN) was examined in a limited group of patients diagnosed with treatment-resistant hypertension. When the two drugs were co-administered, an additional reduction of blood pressure was recorded compared to when received separately, without significant side effects being noted. However, additional long-term studies must be implemented so that the researchers may reach a safer conclusion considering the potential therapeutic efficacy of this combination [75]. Finally, orthostatic hypotension was observed in co-administration of sildenafil with α-adrenergic blockers in some patients who were not stabilized in treatment with these agents [74].

### 5.4. Pharmacokinetic/Pharmacodynamic Interactions

Since sildenafil shares a common metabolic pathway with multiple drugs, potential interactions occur during co-administration. In two placebo-controlled, parallel-group studies, the pharmacokinetics of a 100 mg single oral dose of sildenafil citrate were evaluated during co-administration with multiple doses of two different macrolide antibiotics, known inhibitors of CYP3A4, i.e., azithromycin and erythromycin. The results received indicated that the basic pharmacokinetic values of sildenafil and UK-103,320 were not affected by the presence of azithromycin. On the contrary erythromycin caused a 2.8-fold increase of area under the curve (AUC), a 2.6-fold increase of C_max_ of sildenafil and a 1.4-fold increase in AUC of the major metabolite. Moreover half—life was increased for one hour and this observation was attributed to the inhibition of the CYP3A4 metabolic pathway [76]. The same research group elucidated the pharmacokinetic interactions between sildenafil and protease inhibitors, namely ritonavir and saquinavir. Two unaliased clinical studies conducted on healthy male volunteers, revealed that multiple doses of both drugs increased systemic exposure to sildenafil and the active metabolite without enforcing hemodynamic changes. Particularly, in the case of ritonavir the plasma concentration of sildenafil and AUC was increased by 300% and 11-fold respectively, a combinatory effect attributed to the parallel inhibition of CYP3A4 and CYP2C9 metabolic routes. In the case of saquinavir the differences in pharmacokinetic profile of sildenafil were less prominent compared to ritonavir but also significant. Thus, lessening the recommended dose of sildenafil to 25 mg was considered necessary during co-administration [77]. In another clinical study conducted on healthy male participants, the effect of multiple doses of darunavir combined with ritonavir on the pharmacokinetic properties of sildenafil was examined, hence the first belongs to the CYP3A4 isoenzyme inhibitors. The results indicated that systemic exposure of sildenafil single dose administration of 100 mg was comparable to sildenafil 25 mg dosing at steady—state concentration in plasma of darunavir/ritonavir, (400/100) mg combination, twice per day. In addition, inhibition of metabolic pathways of sildenafil in the presence of darunavir/ritonavir combination minimized the N-demethylation of sildenafil by 20%, thus resulting in a reduced exposure of N-desmethylsildenafil in plasma [78]. Similarly, the impeding of hepatic metabolism of sildenafil was the main cause of the 4.4-fold increase in the AUC of the 25 mg dosage of sildenafil when co-administered with indinavir, in a clinical study recruiting HIV-patients [79]. In these cases, as well as when co-administered with nelfinavir [80], sildenafil did not appear to affect the pharmacokinetics of antiretroviral agents, indicating that it acts as a weak inhibitor of the relevant metabolic isoenzymes.

Similarly, coadministration with multiple doses of cimetidine, another nonspecific inhibitor of CYP3A4, brought about changes in the pharmacokinetic profile of sildenafil. The inhibition of first pass metabolism of sildenafil led to an approximately 50% increase in plasma concentration and an approximately 30% increase in the area under the curve of N-desmethylsildenafil. However, these alterations were not considered to pose any clinical significance. In the same study during concomitant administration of sildenafil with antacin, negligible differences were observed that did not affect the pharmacokinetic data of sildenafil [81]. The results of a randomized, placebo-controlled, double blind crossover study on healthy males demonstrated that the antidepressant fluvoxamine altered the pharmacokinetic parameters of sildenafil as a consequence of CYP3A4 mediated first-pass metabolism inhibition. Indeed, after the single dosing of 50mg sildenafil in the presence of fluvoxamine a high elevation, approximately 40%, in sildenafil systemic exposure was observed while terminal half-life shown extension by 0.5 h [82].

Except from drugs, substances that may affect the first- pass metabolism controlled by CYP3A4 such as grapefruit, which claim a significant inhibitory effect, could also alter the pharmacokinetics of sildenafil. A relevant clinical study conducted on healthy male volunteers exposed that intake of 50 mg sildenafil with grapefruit juice influenced its oral bioavailability causing a 23% increase due to the reduction of its first-pass metabolism and consequent delay in absorption [83]. Surprisingly, the opposite effect on bioavailability was observed when sildenafil was co-administered with pummelo juice. Even though the mechanism of this interaction was not clarified, the rate of sildenafil bioavailability showed a reduction of around 60%, which led to decreased effectiveness [84].

### 5.5. Pharmacokinetic Interactions with Endothelin Receptor Antagonists

Since the treatment options of idiopathic pulmonary arterial hypertension (PAH) are limited, several research groups have focused on determining the pharmacokinetic interactions of endothelin receptor antagonists (ETRAs) with sildenafil. The results of co-administration of bosentan with sildenafil in ten patients with pulmonary arterial hypertension indicated that the maximum plasma concentration and plasma AUC of both sildenafil and its main metabolite were significantly lowered and the clearance of sildenafil showed a rise of about 50%. The possible cause of this interaction is that bosentan is an inductor of expression of CYP3A4, the main metabolic pathway of sildenafil [85]. A corresponding research conducted on healthy volunteers, confirmed that in the presence of bosentan, the systemic exposure to sildenafil was drastically decreased. On the other hand, sildenafil actually augmented the bosentan maximum plasma concentration and AUC by 42 and 50%, respectively [86]. The underlying mechanism for this increase may be attributed to hepatic uptake reduction due to the inhibition of OATP1B1 and OATP1B3 caused by sildenafil [87]. The mutual pharmacokinetic interactions were also determined in the case of macitentan, a recent endothelin receptor antagonist, in the case of concomitant intake. The results exposed that sildenafil did not influence the pharmacokinetics of macitentan and the effect on sildenafil was characterized by minor clinical significance. Furthermore, unlike bosentan, this endothelin receptor inhibitor was reported as an improbable inducer of CYP3A4 and no dose adjustment was suggested during coadministration with sildenafil [88]. Similarly, a two-period crossover study on healthy volunteers reported that no clinically significant mutual pharmacokinetic interaction between ambrisentan and sildenafil or N-desmethyl sildenafil occurred during concomitant intake, therefore combination therapy is well tolerated [89].

### 5.6. Administration to Special Populations

#### 5.6.1. Elderly

The comparison of the data collected from an open-label, parallel-group, single-dose study between healthy young men with an average age of 30 years and healthy men over 65 years old, indicated important variances in pharmacokinetic profile of sildenafil and its main metabolite between the two groups. Specifically, the free sildenafil plasma concentration was increased by 40% in elderly subjects because of greater protein binding that led to lessening of the unbound fraction. The reduced elimination rate in the elderly group was attributed to an increased half-life i.e., about 1.2 h for sildenafil and about 2 h for N-desmethysildenafil. Significant alterations were also detected in the AUC estimation which showed a double rise, as well as in maximum concentration rate which was 60–70% higher for the elderly group compared to young male subjects [90].

#### 5.6.2. Patients with Renal and Hepatic Impairment

The same team elucidated the alterations on sildenafil and its N-desmethyl metabolite pharmacokinetic values and in the case of renal or hepatic insufficiency [90]. The study revealed that men with mild or moderate renal damage compared to healthy subjects did not present significant changes in pharmacokinetic parameters of both sildenafil and metabolite UK-103,320. Important increases in AUC (100%) and C_max_ (88%) of sildenafil highlighted between patients with extensive renal impairment (creatinine clearance <30 mL/min) and healthy volunteers, were influenced by the drastic reduction of sildenafil clearance in those patients [90]. A randomized, 2-phase crossover, open-label study, conducted on fifteen male subjects under hemodialysis, clarified that neither sildenafil nor its major metabolite were cleared when 50 mg of the drug were administered at check points of 2 h prior or post hemodialysis.

In fact, the pharmacokinetic profiles of both sildenafil and UK-103,320 in these patients, were found more similar to those of healthy men than to patients with renal insufficiency [91]. In patients suffering from mild to moderate hepatic cirrhosis, the systemic exposure of parent drug after single administration of 50 mg sildenafil, increased significantly compared to healthy subjects by approximately 80%. In addition, a reduction of about 46% in oral clearance and a higher rate about 47% and 34% in maximum plasma concentration and half-life of the drug respectively, were observed in patients of stable hepatic damage owing to limitation of first-pass effect. An increase in systemic exposure for the N-desmethyl metabolite that reached the 100% compared to parent drug, led to the conclusion that hepatic impairment could influence the elimination rate of UK-103,320 more drastically compared to the main drug. Consequently, for patients with severe renal or liver damage a dose adjustment of 25 mg sildenafil is suggested [90].

#### 5.6.3. Patients with Pulmonary Arterial Hypertension (PAH)

In population pharmacokinetic studies, patients with pulmonary arterial hypertension presented 20–50% increase in levels of mean steady state concentrations of sildenafil in a dose range of 20–80 mg given three times daily. Compared to healthy volunteers the minimum plasma concentrations appeared to be twice as high, indicating reduced degree of clearance and enlarged oral bioavailability. In addition, in patients with PAH receiving 80 mg given three times a day an elevation in oral bioavailability of sildenafil about 43% was observed in comparison to lower doses [92].

#### 5.6.4. Pediatric Populations

Clinical trials have been performed in pediatric populations to define the suitability and pharmacokinetic profile of sildenafil as a treatment option for children burdened with pulmonary arterial hypertension. A randomized 16-week, placebo-controlled, dose-ranging clinical study was conducted on 235 children aged 1 to 17 years (STARTS-1), with main objective of the study the examination of the effect of sildenafil on exercise capacity by measuring the maximum volume of oxygen consumed during exercise. The results after 16 weeks indicated that maximum volume of oxygen consumed by children and hemodynamics during exercise improved with medium and high doses of the drug versus to low doses and placebo. A second long-term extension study (STARTS-II) succeeded the first for the patients who completed the trial. The STARTS-I placebo subjects were re-randomized to low-, medium-, or high-dose sildenafil and an unexpected rise in the death rate was monitored to those who received the highest dose of sildenafil compared to the lowest doses. Evaluation of the pharmacokinetic profile in these trials indicated that body weight index plays the governing role in systemic exposure of sildenafil in children. After single oral administration of 10 mg or 20 mg sildenafil, maximum plasma concentration presented an elevated increase for 70, 20 and 10 kg patients, while T_max_ was estimated at about 1h and did not appear to be affected by body weight [93]. The data collected by these trials caused controversy between European Medicines Agency (EMA) and FDA, with the first proposing the use of low-dose sildenafil in pediatric populations and the second to subject a recommendation against with recommended use only in situations where the risk-benefit balance is acceptable [94].

In an open-label study conducted on 36 neonates with persistent pulmonary hypertension of the newborn (PPHN) or neonatal hypoxemia, after intravenous administration of sildenafil within 3 days after birth and for a period of seven days, significant variations in pharmacokinetic parameters were recorded. Because of lower levels of protein binding in neonates, total volume of distribution of sildenafil was significantly higher compared to adults leading to a prolongated half-life of 6.8 h, and typical terminal elimination half-life of 56 to 48 h from day one to seven. The clearance of sildenafil presented a 3-fold increase from day after birth until the end of the study. This clearance maturation was attributed to the rapid developmental alterations in the expression of CYP enzymes in neonates and additional research needs to be implemented for the determination of the appropriate dose and efficacy of sildenafil in newborn patients [95].

## 6. Alternative Administration Schemes

Statistically important reduction in absorption rate is observed whereas sildenafil is co-administered with high fat meal (delayed t_max_ by 1 h in fed state compared to 30–120 min t_max_ in fasted state, 90% confidence intervals CI: 0.7 ± 1.5 using ANOVA appropriate design) as well as in mean C_max_ (−29% in fed state, 90% CI: 19 ± 38) [60]. The combination of food effects with the diminished bioavailability due to extensive first path metabolism [60], has led contemporary researchers to focus on upgrading the existing oral dosage formulations [96,97,98,99,100] and even apply alternative routes of administration i.e., intranasal, transdermal and intravenously [101,102,103].

### 6.1. Oral Administration

The oral formulations being the frequent dosage form are classified as tablets, chewable, capsules, suspensions and liquids. Tablets and capsules provide advantages like stability, accurate dosing, easy manufacturing, restricted packaging size and easy handling. On the other hand, they may face imperfect patient compliance owing to swallowing difficulties and reduced bioavailability owing to first pass metabolism. In order to avoid the said drawbacks, researchers focused in developing orodispersible formulations predominantly absorbed by sublingual and supralingual routes.

#### 6.1.1. Orally Disintegrating Tablets (ODT)

There exist a variety of orodispersible tablets in the market, due to their ability to disintegrate easily in the mouth [104]. Orodispersible tablets are orally disintegrating tablets commonly referred to as mouth-dissolving tablets, rapid-dissolving tablets, fast-disintegrating tablets and fast-dissolving tablets. They disintegrate within 3 min of contact with a tiny volume of saliva in the oral cavity before swallowing [96]. This formulation offers high patient administration compliance excluding the need of water intake while also retaining the advantages of common tablets i.e., stability, accurate dosing, easy manufacturing, limited packaging size and easy handling [96]. The disadvantages of the formulation are the high first pass metabolism, the bitter taste and the considerable feeding interaction [105]. Orodispersible tablets of sildenafil have been marketed since 2009 bearing the proprietary name of Vizarsin in doses of 25, 50, 100 mg.

In 2010 the Pfizer Clinical Research Unit in Singapore, conducted a bioequivalence study of a sildenafil ODT formulation in order to test posology combined with water and to evaluate the pharmacokinetic effects after a high-fat meal intake. Four years later the research team announced that the formulation when tested in the absence of water intake was found bioequivalent to the marketed sildenafil film-coated oral tablet, hence for 90% confidence interval (CI) the ratio of geometric means of C_max_, AUC_0–1_, and AUC_0–last_ were documented as contained within acceptance limits (80–125%). However, when the same ODTs were co administered with water for the 90%CI, the C_max_ was estimated outside of acceptance range (79.76–92.78) and therefore deemed non bioequivalent although the AUC did satisfy the criteria [105]. In addition, considering these results of the food-effect, sildenafil ODT should be administered on an empty stomach in order to eliminate the drug-food interaction ((https://clinicaltrials.gov/ct2/show/NCT01254396) (www.clinicaltrials.gov) (accessed on 11 April 2021)).

The Singh group investigated in 2014 several stabilizers (PVP K-30, Crospovidone and Kyron T-134) in pre-formulation concentrations and also common favor/color excipients (calcium carbonate, aspartame, aerosil, magnesium stearate, talc, mannitol). The FT-TR spectrums showed that sildenafil citrate did not interfere with the polymers and an optimum ODT formulation containing Crospovidone and Kyron T-134 with 96.364% drug release within 15 min was reached [106]. According to another successful bioequivalence/bioavailability open-label, randomized clinical study, sponsored by Pfizer in 2016 on healthy Chinese men, a 50-mg monodose of sildenafil citrate ODT administered with and without water intake was compared against the equal dose of the commercial film-coated tablet (Viagra; Pfizer Inc, Dalian, China) [107]. The disadvantage though of the said formulations is inconveniency owed to bitterness. Answering the challenge Wang and Sun isolated sildenafil free base and then prepared a pure bulk salt of the API with the artificial sweetener acesulfame (Acs), employing an equimolar acid-base reaction and obtaining products of remarkable thermic stability (up to 185 °C in TGA) and low hygroscopicity (negligible moisture uptake even at 95% RH). The dissolution rate though of these sildenafil—Acs complexes at 37 °C, was found lower than the intrinsic dissolution rate of sildenafil citrate i.e., 7.6 × 10^−3^ μmol·min^−1^·mL^−1^ and 1.6 × 10^−2^ μmol·min^−1^·mL^−1^, respectively [108].

#### 6.1.2. Oro-Dispersible Film (ODF)

The ODF formulations are thin film composites placed directly on the tongue surface, their advantage being the rapid local disintegration without the assistance of chewing and/or presence of water [109]. ODF formulations have been developed in accordance with the patent contents EP 168,937,4 and WO 2014/049548 [110] by IBSA (Lugano, Switzerland) at doses of 25, 50, 75, and 100 mg of sildenafil. It is indicative of the formulation that the ODF contains 105.3 mg of sildenafil citrate to meet equivalence criteria of sildenafil 75 mg [97]. The excipients contained in the formulation were maltodextrin and the plasticizer propylene glycol. Both sublingual and supralingual administration routes were examined for the ODF formulation presenting a similar pharmacokinetic and safety profile.

#### 6.1.3. Sublingual Delivery Systems

The Sheu group developed a sublingual delivery system prepared by various granulated sprays adsorbed onto a silicate substrate. The optimal formulation contained sildenafil citrate (0.5 mg) in a lipid vehicle of propylene glycol, Florite^®^ R as an adsorbent, Cyclocel^®^ as the binder and Ac-Di-Sol^®^ as the disintegrant [98]. The investigation of pharmacokinetic studies was performed on rabbits (3.0–4.0 kg) at the dose of 50 mg demonstrating absolute bioavailability of 39.7%, T_onset_ 5.5 min, and active duration for longer than 3 h; however, the C_max_ was found 3596.4 nM i.e., above the targeted threshold [98]. The optimized formulation demonstrated higher absolute bioavailability, 90.24%, T_onset_ 1.9 min, duration 62 min and C_max_ within the bioequivalent range [98].

#### 6.1.4. Oral Pediatric Suspensions

Another challenging issue is the preparation of safe and stable liquid formulation for pediatric use [111]. Provenza studied two liquid formulations of sildenafil citrate at pH 4 i.e., an oral re-dispersible suspension and a transparent non glucose solution. From the physicochemical and microbiological point of view the suspension was stable for 90 days (no detectable changes in color or odor at controlled temperatures/stable Newtonian behavior) while the uniformity of sildenafil citrate content was above 90%. On the other hand, solution remained stable for 30 days when it was stored at 25 °C, but at 4 °C the API concentration was eliminated to under 90% after day 15 [112]. These findings suggested that sildenafil citrate oral suspension might be used for the treatment of neonatal persistent pulmonary hypertension. In 2015 at Pfizer’s Clinical Research Unit, in Bruxelles, researchers tested the equivalence of the three sildenafil formulations intending for pediatric patients with PAH namely: intact tablet (A), sildenafil 20-mg crushed tablet mixed with apple sauce (B) and sildenafil 20-mg suspension (C). The bioequivalence was established although the suspension showed a 15% decrease in C_max_ which was not considered clinically relevant hence the evaluated AUCs assured the main efficacy response [113].

#### 6.1.5. Chewable Tablets

In order to avoid the disadvantages of the film-coated tablet i.e., poor patient compliance owing to swallowing difficulties Yoo et al. prepared chewable sildenafil tablets. In detail, C_max_ and AUC_last_ geometric mean ratios for 90% CI, indicated bioequivalence between the chewable sildenafil tablet 0.933 (0.853–1.021) and the marketed one 1.034 (0.964–1.108), within the Korean bioequivalence criterion of 0.80 to 1.25 [99]. The clinical study was conducted in 60 healthy male volunteers and concluded that the chewable tablet showed similar pharmacokinetic properties to the market product and therefore can be an alternative to sildenafil for the treatment of erectile dysfunction, providing better compliance [99].

#### 6.1.6. Dry Foam Tablets

Dry foam technology was developed following the intention to screen the optimum ingredients in precise concentration, aiming to enable a faster and more efficient dissolution rate by avoiding API agglomeration and floating of the non-wetted API particles [114,115]. Sildenafil citrate was formulated suspended in sodium dodecyl sulphate solution followed by the addition of diluents (mixture of maltodextrin and mannitol) into paste [116]. The formulation was then passed through a nozzle of spray bottle in order to obtain a homogeneous smooth foam which was dried in vacuum and passed through the sieve no. 18 to obtain dry granules. The material was then mixed with croscarmellose sodium, magnesium stearate and compressed into tablets [117]. The final formulation was administrated orally to male Wistar rats and resulted in systemic bioavailability of 1.5 and 1.9 times higher than that of commercial version of sildenafil film-coated tablet and sildenafil powder of 20 mg/kg sildenafil, respectively [100]. Comparing to the sublingual delivery system mentioned above the bioavailability of the dry foam tablets was lower [98] due to the rich blood supply of the sublingual vessels and the hepatic first pass metabolism escape or due to the physiological variation between the animal species.

### 6.2. Intranasal Microemulsions

Intranasal (IN) administration has the main advantage of avoiding first pass metabolism and thus offering rapid absorption [118]. Despite this major benefit, IN route has some significant limitations like poor permeability of drug across nasal mucosa and mucociliary clearance. This might explain the fact that there exist no marketed nasal spray formulations of sildenafil although there a patent for nasal administration of sildenafil has been filed since 1998 [101]. Exploring the feasibility of IN formulation Elshafeey et al., 2009 proposed an alternative solution in order to address the problem of low solubility of sildenafil citrate. As excipients for the preparation of the oil phase isopropyl myristate, miglyol, labrafil^®^ M and oleic acid were tested and the optimal developed formulation was composed of oleic acid/Labrasol/Transcutol/H_2_O (8.33:33.33:16.67:41.67%). The research group achieved a sevenfold increase in solubility, significantly higher C_max_, shorter T_max_ and relative bioavailability of sildenafil citrate 112.89%.

In 2011 another research group also studied an oleic acid-based microemulsion system for rapid-onset intranasal delivery of sildenafil. The microemulsion featured a droplet size 20 nm and consisted of 40% oleic acid, 10% H_2_O, and 50% Tween 80: ethanol (at a 1:4 weight ratio) [119]. The sildenafil citrate solubility in the emulsion was estimated 124 mg/mL with the optimal nasal formulation demonstrating absolute bioavailability 24%, excellent time of onset, duration 3h, and most importantly, C_max_ did not exceed the toxic concentration [7,119].

### 6.3. Inhalables of Controlled Release

The oral administration regimen of sildenafil, as therapy against pulmonary arterial hypertension, causes systemic drug exposure and unwanted side effects like headache, flushing, dyspepsia, back pain, diarrhea, limb pain, myalgia cough etc. [120]. Beck-Broichsitter et al. observed that, the same dose of nanoencapsulated sildenafil resulted in prolongation of the pulmonary vasodilatation. They used submicron particles (mean size of ~200 nm) delivered to the airways by a Micro-Sprayer^®^ which was integrated into the inspiratory tubing system with the tip of the nebulizer approximately 1 cm above the tracheal bifurcation. The formulation was synthesized from biodegradable PLGA and P(VS-VA)-g-PLGA with a theoretical sildenafil loading of 5 wt.% by modified solvent evaporation [121,122]. The utilization of the charge-modified branched (i.e., P(VS-VA)-g-PLGA) rather than the linear polyesters (i.e., PLGA) significantly advanced the release profile, due to the increased charge-density within the polymeric submicron particles, facilitated the relevant electrostatic interactions with sildenafil.

### 6.4. Transdermal

A lot of research effort has been devoted on transdermal drug delivery systems for nanosized sildenafil particles [123] to improve drug solubility and bioavailability, while achieving high drug loading [124]. The nano comminution of starting material is a credible, scalable technique to increase the available contact surface and dispersion capacity of the BCS II APIs [125]. The Elnaggar group used a modified high-shear homogenization technique to fabricate sildenafil citrate-loaded nanostructured lipid carriers (NLCs) and solid lipid nanoparticles (SLNs) achieving entrapment efficiency of 96.7% and 97.5%, respectively. Results revealed that SLNs and NLCs vehicles were optimized in the nanometric range between 180 and 100 nm, respectively [102]. The transdermal permeation of sildenafil nanocarriers significantly enhanced the initial available concentration followed by controlled release, with promising implications for faster onset and longer drug duration [102].

### 6.5. Intravenous

Liposomal formulations are also used as carriers for targeted delivery and amelioration of toxicity [126]. The Li research group in 2019 used glucuronic acid (GlcA) to formulate liposomes of size 90 nm containing S100, dioleoyl phosphoethanolamine [DOPE], cholesterol, and DSPE-PEG2000-GlcA, loaded with sildenafil. When intravenously injected to rats, the result was 1-fold increase of GlcA-Lips uptake. These formulations demonstrated longer blood circulation time of GlcA-Lips and increased ability to target pulmonary arteries by 1-fold after 8 h administration [103]. In Table 2 and Table 3 the advantages and disadvantages of the various available compositions and the indicative marketed formulations respectively, are summoned and displayed.

## 7. Analytical Strategies for the Determination of Sildenafil in Pharmaceutical and Biological Matrices

A literature survey reveals that several analytical protocols have been published up to now for the determination of sildenafil in pharmaceutical dietary products, herbal products and biological matrices. In this section, we describe the analytical strategies developed for the analysis of the drug, which are classified into two main categories oriented to pharmaceutical and biological applications.

### 7.1. Pharmaceutical Applications

The commercial formulation of sildenafil is available as film-coated tablets containing 25, 50 or 100 mg API. On the other hand, several studies shown that sildenafil has been illegally used in certain dietary supplements, herbal medicines and alcoholic beverages. The high consumption of the substance as a medicine or dietary supplements, around the world, indicates the importance of developing different analytical approaches for monitoring not only the quantity but also the authenticity of products.

**Figure 4:**
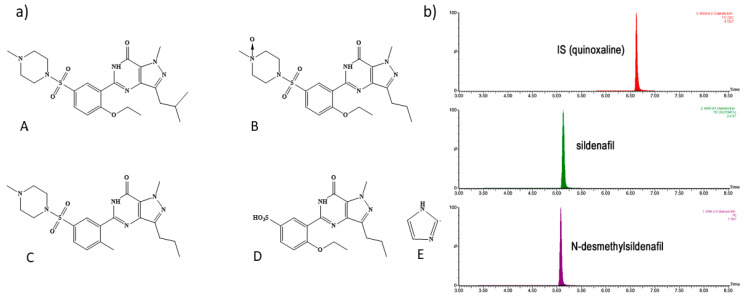
(**a**) Chemical structure of sildenafil impurities A, B, C, D and E.; (**b**) MRM chromatogram of N-desmethylsildenafil, sildenafil and quinoxaline (internal standard) adopted with permission from [30]. 2015 Elsevier.

Officially, the identification of the raw material, according to European Pharmacopoeia [133], is achieved by IR spectroscopy while the API’s impurities A, B, C, D, E (Figure 4a) are quantified by a TLC method (silica gel plate F254 2-10 μm) using as mobile phase: concentrated ammonia, ethanol (96%), ethyl acetate, methylene chloride (1:20:30:50 *v*/*v*/*v*/*v*). At the same time, sildenafil citrate and its related substances are quantified by HPLC-UV method at 290 nm with an end-capped C_18_ (150 × 3.9 mm, 5 μm) column, thermostated at 30 °C and a mobile phase consisting of 17/25/58 17% acetonitrile/25% methanol and 58% water solution with 0.7% (*v*/*v*) triethylamine solution adjusted to pH 3.0 ± 0.1, with phosphoric acid. In addition to the standard method, there are many analytical techniques for the determination of sildenafil citrate in herbal, dietary products or pharmaceutical formulations that will be summarized in the current review. The determinations can be classified into three main categories: (i) optical, (ii) electroanalytical, (iii) chromatographic methods.

#### 7.1.1. Optical Methods

Electrochemical and spectroscopic methods are suggested as an on-field screening tool, suitable for use in cases where an analytical inspection of sildenafil is required in a large number of products suspected of being adulterated. Such methods are simple, fast, highly efficient and require little sample preparation.

##### Spectrophotometric Methods

Due to the chromophore groups (extended coupling of single, double bonds and substituents with n-linked electrons) presented in the sildenafil molecule, the UV absorption band of the substance exhibits two peaks at 220 and 292 nm, respectively. Thus, many analytical UV methods were used for the quantification of sildenafil at dietary supplements [134] or pharmaceutical formulations [135]. In 2001, a flow injection analysis (FIA) with UV detection was applied by Altiokka et al. [136] for the determination of sildenafil in Viagra^®^ tablets (50 mg). The flow solution consists of 0.2 M phosphate buffer at pH 8, having 10% MeOH (flow rate of 1 mL min^−1^) and detected at 292 nm. In the same year, the molecular interaction between sildenafil as electron donor and iodine; 7,7,8,8-tetracyanoquinodimethane; 2,3-dichloro-5,6-dicyano-1,4-benzoquinone; tetracyano-ethylene; 2,4,7-trinitro-9-fluorenone; chloranilic acid; chloranil and bromanil as acceptors was investigated spectrophotometrically. Beer’s law was obeyed in a concentration range of 10–260 mg/mL [137].

Ion-associate complexes of sildenafil citrate with bromocresol green and with chromoxane cyanine R in aqueous acidic buffer were applied for its extractive spectrophotometric detection. The complex species were extracted in chloroform phase and then quantified at 415 and 460 nm, respectively. The colored complexes were found to be stable up to 35 ^o^C [138]. Another spectrophotometric determination of sildenafil citrate in pure form and in pharmaceutical formulations using some chromotropic acid azo dyes are presented by Issa et al. The method based on ion-association of the substance with two groups of monochromotropic acid azo dyes. The measurements were performed at 540, 520, 540, 570, 600, and 575 nm after the methylene chloride extraction [139].

In aqueous solutions, sildenafil presents a very low fluorescence emission and limited linear range. In the presence of a cationic (hexadecyltrimethylammonium bromide) and an anionic surfactant (sodium dodecyl sulfate) a great fluorescence enhancement was observed by Wang et al. [140]. Sildenafil and surfactants were quantified at 435 nm and 415 nm emission wavelengths in both methods, respectively. The methods have been successfully applied in the analysis of bulk drugs, tablets and herbal medicines.

The corresponding research team in a similar study proposed a sensitive Amberlite XADTM resin coated with a surfactant for sildenafil spectrofluorometric detection [141]. Thus, the retention capacity of micellar coated XADTM resin by sildenafil was studied and the eluate obtained was measured with a spectrofluorometer at excitation and emission wavelengths of 350 and 430 nm, respectively.

##### Other Spectroscopic Methods

An Opto Trace Raman (OTR) 202, as Surface Enhancing Raman Spectroscopy (SERS) active colloids, was used to detect sildenafil in alcoholic drinks. The results demonstrated that the Raman enhancement factor of OTR 202 colloids reached 1.84 × 107 and the limits of detection (LODs) at 0.1 mg L^−1^. Moreover, the SERS peaks of 645, 814, 1235, 1401, 1530 and 1584 cm^−1^ could be identified as characteristic sildenafil’s peaks [142]. Two other spectroscopic methods, a near-infrared (NIRS) [143], and a Raman method [144] were suggested as rapid screening techniques for Viagra^®^ tablets. The methods can be used to check the homogeneity of a batch and to distinguish genuine Viagra^®^ tablets from counterfeit and imitations ones.

#### 7.1.2. Electroanalytical Methods

The electrochemical determination of sildenafil citrate was based in the finding that in aqueous solutions, containing 30% (*v*/*v*) acetonitrile, the unprotonated form of the piperazine ring of the substance is oxidized on a glassy carbon electrode between pH 2 and 8 [145]. Two potentiometric sensors responding to sildenafil citrate are described, characterized, compared and used by Hassan et al., respectively, for the drug assessment [146]. The sensors are based on the ion-association complexes of API (cation) with tungstophosphate and reineckate (anions), as electroactive in plasticized poly(vinyl chloride) membranes. Both sensors were demonstrated fast near-Nernstian response with detection limits of 0.53 and 0.67 μg mL^−1^ (over pH 3–6) for both complex, respectively.

On 2008, a simultaneous determination of sildenafil in the presence of paracetamol and carvedilol was performed, by Baranowska and her research team, using cyclic voltammetry via a glassy carbon electrode (GCE) as working electrode. An Ag/AgCl/KCl (sat.) electrode served as the reference electrode, and a platinum wire as the auxiliary [147]. Alternatively, three types of monocrystalline diamond (natural 1 µm, synthetic 50 µm and synthetic 1 µm) were used for the design of diamond paste electrodes appropriate for the determination of sildenafil citrate (Viagra^®^) with square wave voltammetry. It was found that the API yielded a peak at about +0.175 ± 0.025 V (versus Ag/AgCl) for all the electrodes [148].

Another rapid and simple electrochemical analysis of the substance was developed using a screen-printed glassy carbon electrode (SPGCE) [149]. Initially, investigations were undertaken using cyclic voltammetry to characterize the redox behavior at the SPCE. The current response was evaluated with respect to the drug composition, pH of the supporting electrolyte, potential and scan rate. The limit of detection was found to be 5.5 × 10^−8^ mol L^−1^. Meanwhile, a flow injection analysis (FIA) with multiple pulse amperometric detector was used for the determination of sildenafil citrate (Viagra^®^) in various pharmaceutical formulations. The method based on the application of three sequential potential pulses as a function of time. API was detected at 1.6 and 1.9 V by two different irreversible oxidation processes. The third potential pulse (1.0 V) was applied for the regeneration (cleaning) of the surface of the boron-doped electrode [150].

Finally, the determination of sildenafil citrate on integrated three-electrode screen-printed sensor (carbon working electrode, auxiliary and a silver reference electrode) was developed on 2019 by Sasal et al. Differential-pulse voltammetric measurements were carried out in 0.15 mol L^−1^ acetate buffer of pH = 5.0 ± 0.1 [151].

#### 7.1.3. Chromatographic Methods

Simple, fast and sensitive high-performance liquid chromatography (HPLC) methods have been developed and validated for the determination of sildenafil citrate in pharmaceutical preparations. The methods refer to reverse phase chromatography and their conditions are summarized in Table 4 [152,153,154,155,156,157,158]. The parameters evaluated were the detection wavelength, the type and amount of the organic modifier, the salt concentration and the pH of the mobile phase. In most cases, the mobile phase used consisted of acetonitrile/water, since this particular organic solvent improve peak ‘s symmetry and reduce the elution time of the API.

Among the HPLC methods developed, a special mention was made to a magnetic solid phase extraction technique, intended to pre-concentrate the API. The method uses a new magnetic nano-diamond/graphene oxide hybrid material as a sorbent [154]. Another remarkable study was performed by using a reversed phase monolithic silica column [152], whereas the determination of sildenafil citrate and its related substances in the commercial products and tablet dosage form was suggested by Daraghmeh et al. [153].

#### 7.1.4. Specifications in an Assessment Report

##### API’s Specifications

Generally, specifications for sildenafil citrate as bulk substance in an assessment report include tests for appearance, clarity and color of the solution, identification, citrates and their content, water content, melting point, residue on ignition, sulphated ash, heavy metals, purity, assay, particle size and residual solvents [159].

##### Stability of API

Stability studies of the active ingredient must be carried out according to ICH [160] guidelines for real time (25 °C/60% RH), intermediate (30 °C/65% RH) and accelerated conditions (40 °C/75% RH). Data for several commercial scale batches must to be collected for 60 months for both real time and intermediate conditions, and for 6 months at accelerated conditions. No trends should to be found at long-term and accelerated conditions for the tested parameters. In addition, results from the forced degradation studies should prove that sildenafil citrate degrades under stress conditions (acidic and alkaline conditions, oxidation, UV irradiation, light and thermal treatment) to the known impurities. No unknown impurities should to be detected during stability testing.

##### Specifications of Tablet Formulations

The tablet specifications for sildenafil are standard and refers to suitable limits for appearance, average mass, resistance to crushing, disintegration time, loss on drying, identity of sildenafil (HPLC), sildenafil citrate (UV) and citrate, identification of colorant (titanium dioxide and iron oxide), assay (HPLC), uniformity of dosage units (HPLC), purity (HPLC), in-vitro dissolution (with UV evaluation) and microbiological purity [159].

##### Stability of Tablets Formulation

Stability studies must be carried out under ICH conditions of 25 °C/60%RH (long term), 30 °C/65%RH (intermediate) and 40 °C/75%RH (accelerated). In addition, photostability testing must be performed according to the “Note for Guidance on the Photostability Testing of New Active Substances and Medicinal Products” [160]. The results from stability studies, including photostability testing, should demonstrate that tablets are stable and no significant changes in tested parameters were observed during the storage.

### 7.2. Bioanalytical Applications

#### 7.2.1. LC-MS/MS

The determination of sildenafil in biological samples is a difficult task since these matrixes are quite complex and contain moderate-to-high levels of proteins. The analytical pipeline typically consisted of a sequence of sample treatment steps—prior to analysis—aiming to minimize the matrix effects, enhance the method selectivity and make the sample compatible with the analytical technique avoiding its contamination [161,162,163]. Established sample preparation protocols—mainly utilized in bioanalysis—involve sample dilution (mostly performed in urine analysis), protein precipitation, liquid-liquid extraction (LLE), solid phase extraction (SPE) and others and combination of the former as well.

In the last decade, various bioanalytical methods have been reported in the literature dedicated to the analysis of sildenafil [164,165,166,167,168,169,170,171,172,173,174,175,176,177,178,179,180,181,182,183,184,185,186]. An overview is presented in Table 5 [30,123,136,137,138,139,140,141,142,143,144,145,146,147,148,149,150,151,152,153,154,155,156,157,158,159]. In these reports, liquid chromatography coupled with mass spectrometry has become the technique of choice offering highly selective and sensitive determinations. Moreover, the LC-MS/MS technique has been considered as the “gold standard” for the screening and identification of erectile dysfunction drugs and their illegal analogues in adulterated food supplements and sexual performance enhancement products [182,183,184].

An interesting LC-MS/MS approach has been published by the research group of Cheng for the determination of sildenafil and its active metabolite N-desmethylsildenafil in human plasma [164]. The plasma samples after protein precipitation with acetonitrile were centrifuged and diluted 4-fold with water prior to LC-MS/MS analysis. Using a sample volume of 100 μL, the sensitivity of the method was satisfactory for the determination of the analytes in the examined samples and to support the clinical pharmacokinetic study. Satisfactory linearity in the range of 2–1000 ng mL^−1^ was achieved where the analytes were detected at multiple reaction monitoring (MRM) mode offering high selectivity and sensitivity. The analytical data were processed to correlate the pharmacokinetic profile of sildenafil of healthy male volunteers with their nutritional content of taken foods in terms of fat, carbohydrates and proteins. The results revealed that food affect the absorption of the drug and its metabolite by delaying the T_max_ and diminishing the C_max_ values.

Three different LC-QTOF-MS/MS approaches for the analysis of four phosphodiesterase type 5 inhibitors–including sildenafil–in biological fluids have been published by the Bakirdere research group [166,168,170]. A simple dilution step was followed by the researchers for the determination of sildenafil and its analogues tadalafil, vardenafil and avanafil in urine samples [170]. The accuracy of QTOF-MS/MS enabled the authors to elucidate the fragmentation pathways of the analytes. Adequate linear ranges between of 5 to 1000 ng/g were obtained for all studied drugs while the matrix effect was less than 10 %. The stability of the target analytes was assessed in simulated gastric fluid where no degradation of the compounds was observed. In the same year, the authors continued their research on this topic by developing a dispersive magnetic solid-phase extraction for the analysis of the specific compounds in human plasma and urine [168]. Although that almost identical LC-MS/MS conditions have been utilized with their previous assay [170], they further synthesized the citric acid coated magnetite nanoparticles.

The manufactured sorbent was characterized by X-ray diffraction and FT-IR spectroscopy. All factors affecting the extraction efficiency of the drugs were investigated and optimized using the one-variable-at-a-time and Box-Behnken experimental design approaches. A good fitting of the experimental data with the predicted ones was observed resulting to a coefficient R^2^ higher than 0.96.

The authors concluded that the extraction performance of the compounds were improved at elevated sorbent amount with minimum eluent volume. From a green analytical chemistry point of view the analytical eco-scale of the method was estimated to be 94, which is superior compared to the other published approaches. A year later, the same group of authors exploited the isotopic dilution strategy in combination with dispersive magnetic solid phase extraction to isolate the analytes from human plasma and urine [166]. The effect of the equilibration period of the blended composition was studied up to 3h with no significant changes after the first hour. One the advantages of the proposed scheme was the remarkable improvement of method accuracy and precision even when complicated matrixes are analyzed.

In 2016, Rashid and Ahsan published a sensitive LC-MS/MS analytical method for the simultaneous determination of sildenafil and rosiglitazone in rat plasma [174]. An efficient sample preparation based on protein precipitation with methanol was followed prior to the analysis. The protonated ions [M+H]^+^ (ESI (+)ve mode) and their prominent fragment ions were used as precursor and product ions for the quantitation of the analytes. Optimal separation was obtained using a gradient elution with water and methanol containing 0.1 % formic acid at a flow rate of 0.25 mL min^−1^. The researchers concluded that the pharmacokinetic profiles of sildenafil alone and of both drugs the AUC_(0–13h)_ and the elimination half-lives were significant different indicating that co-administration of rosiglitazone increases the amount of sildenafil in the body and prolong the elimination half-life of sildenafil. A quite similar sample preparation has been demonstrated by El-Bagary et for the isolation of the sildenafil from human plasma matrixes [181]. The main objective of the proposed research was the development of an UHPLC-MS/MS in order to determine the safety margins for drug combinations by analyzing sildenafil and nitric oxide releasing compounds. The approach was validated according to the FDA guidelines for bioanalytical methods. Potential future perspectives on the developed assay include its application in bioequivalence studies and in health care in order to manage patients suspected of receiving sildenafil and nicorandil and arginine.

Sildenafil is metabolized in the liver by CYP3A4 and CYP2C9 cytochromes and converted to its active metabolite, N-desmethylsildenafil. A simultaneous determination of both compounds have been reported by Simiele et al. [30]. Electrospray ionization in positive mode was used to produce the quasi-molecular ions of the analytes and further MS/MS fragmentation. Depending on the structure of the compound, the characteristic ions appeared at different fragmentor voltage. The method thoroughly validated according to FDA guidelines while the lower limit of detection and quantitation achieved were 1.95 and 3.9 ng mL^−1^ respectively. A representative MRM chromatogram of analyte and internal standard is depicted in Figure 4b.

A group of five erectile dysfunction drugs (mirodenafil, sildenafil, tadalafil, udenafil and vardenafil) and their selected metabolites were determined in hair using an LC-MS/MS method [177]. After the optimization of the method parameters the samples were incubated in acidic methanol followed by solid phase extraction using C_18_ mixed mode strong-cation exchange polymeric cartridges. The separation of the analytes were carried out under reversed phase conditions on a Agilent Poroshell 120 EC-C_18_ analytical column using mobile phase a mixture of water and acetonitrile both acidified with 0.1 % formic acid. A hydrophobic sorbent-based SPE has been utilized for the treatment of human plasma prior to quantification of the drug of interest with LC-MS [184]. The authors basified the biological sample at pH 9.0 in order to enhance the retention of the sildenafil molecule on the SPE sorbent. The quasi molecular ions ([M+H]^+^) of 475.3 and 372.3 m/z were selected for the monitoring of sildenafil and the internal standard trazodone. The method offered adequate sensitive of 5 ng mL^−1^ and acceptable accuracy being in the range of 87.9–110.1%.

Liquid extraction has been applied in two analytical LC-MS/MS protocols developed for the determination for the analyte in dried blood spot (DBS) [182] and human plasma [183]. In 2014, a DBS-based LC-MS method has been reported by the Jimenez and his co-workers [182]. DBS offers several advantages compared to the conventional whole blood collection as it is less invasive sampling method and provides simpler sample storage and transfer without freezing [185]. Since children with pulmonary hypertension have lower metabolic rate instead of healthy adult males bioaccumulation of consecutive doses of sildenafil up to hazardous levels could be occur. In order to support the pharmacokinetic study, a LLE protocol using methyl *tert*butyl ether has been developed for the analysis of sildenafil and N-desmethylsildenafil by LC-MS/MS [183].

#### 7.2.2. HPLC-UV

The chromatographic separation of a group of drug (sildenafil, avanafil, apomorphine, trazodone, yohimbine, tramadol and dapoxetine) was investigated on two different stationary phases namely core-shell particulate and monolithic material [167]. Ion-pair reagent (sodium octanosulfonate) has been employed as mobile phase additive to enhance the retention and the separation efficiency of the compounds in the studied analytical columns. The usage of ethanol as organic modifier is considered to be advantageous compared to acetonitrile from a green analytical chemistry point of view but broader peaks and reduced separation efficiencies were obtained. Additionally, monolithic stationary phases offered faster analysis (9 min) compared to core-shell ones (16 min) while both columns have been successfully applied for the analysis of the above compounds in pharmaceutical dosage forms and human plasma.

An interesting approach has been developed by the research group of Strach for the quantitation of sildenafil in rat plasma [176]. The authors exploited a typical LLE step using a mixture of ethyl acetate/hexane, 30/70 *v/v* and then the analyte was back-extracted into an acidic aqueous phase (0.1 M H_2_SO_4_) in order to improve further the clean-up the biological sample. The specificity of the described method was proved by analyzing blank rat plasma while the accuracy was adequate with recoveries higher than 80%. Long-term stability studies of the treated plasma samples (−80 °C) revealed that the drug was stable up to 60 days. The appropriateness of this method was demonstrated in a pharmacokinetic study in rats after a single intravenous and oral administration of sildenafil. On the same fashion, a back-extraction step using acidic medium (5% *v/v* HClO_4_ aqueous solution) was followed by Alkhawaja and his colleagues [175]. In order to assess the linearity of the method calibration curves was constructed over a period of six consecutive days in the range of 2–200 ng mL^−1^. Statistical treatment the regression equations led to 1/x weighted calibration curves. According to the authors the back-calculated concentration each calibration levels was less than 15% of the nominal concentration.

A two-step extraction for the analysis of sildenafil, vardenafil and sildenafil in human plasma was reported [186]. These include ionic liquid-based dispersive liquid liquid microextraction followed by back-extraction of the analyte in acidic medium. A systematic optimization of the method parameters was performed while the final procedure comprises the extraction of sildenafil by adding 20 μL of 1-octyl-3-methylimidazolium hexafluorophosphate, 20 μL methanol and 300 mg NaCl in 960 μL sample. The analyte was then back-extracted in 10% *v/v* acetic acid and analyzed by HPLC-UV. Compared to other methods, sildenafil was monitored at 254 nm instead of 230 nm [171,172,175,176,185].

#### 7.2.3. Voltammetry

An integrated screen-printed electrode was developed for the analysis of sildenafil in pharmaceutical formulations and biological samples [151]. The voltammetric measurements were carried out using a glassy carbon electrode. The sensitivity of the method was studied at pH values of 1–10 while higher signals were obtained using acetate buffer. The accuracy of electrochemical method was investigated using standard addition method in human serum. A quite similar method has been implemented by Farghali et al. [178]. A gold nanoparticles-modified screen-printed electrode was fabricated by electrodeposition. The selectivity of the electrochemical sensor against to ascorbic acid and uric acid was examined. Although the proposed assay exhibited excellent analytical figures of merit the application of the method in simulated only human urine could be a shortcoming of the approach.

## 8. Discussion

Sildenafil citrate features low solubility (at neutral pH conditions 0.02 mg/mL), high permeability and ampholytic behavior, while the base form is practically insoluble in distilled water. The API is chemically synthesized based on the route selection of pyrazolo [4,3-d]-pyrimidines, including the isolation hurdle step of formation of a double salt as an intermediate. Pharmacologically, the drug is a potent selective, reversible inhibitor of phosphodiesterase type 5 (PDE5) and has received regulatory approval for the treatment of erectile dysfunction and pulmonary arterial hypertension. The beneficial vasodilatory effect also causes a moderate reduction of both systolic and diastolic blood pressure. Since orthostatic hypotension is observed in co-administration of sildenafil with α-adrenergic blockers, coadministration with organic nitrates is contraindicated. A wealth of clinical findings indicates that the pharmacological action of sildenafil response is perceptible at times beyond the established therapeutic window. Sildenafil is accumulated and maintained in vascular smooth muscle cells of the penis due to its high PDE5 affinity. Upon cGMP increase, sildenafil converts the catalytic center of the PDE5 enzyme into a higher affinity structure preventing cGMP hydrolysis. Recent research demonstrates that the high selectivity of PDE5 for sildenafil can be achieved not only by activation, due to the increase of cGMP concentration, but also by the independent regulation of PDE5 in the non-activated form.

Sildenafil is highly absorbed, however, the resulting absolute bioavailability does not exceed 41%, due to the extensive first-pass metabolism effect. A maximum plasma concentration C_max_, under fasting conditions, is achieved within 30 to 120 min. The clearance capacity has been estimated to 41 L/h and the half-life of the total clearance is determined at 3.7 h. The main elimination root after intravenous or oral administration for all metabolites is through feces (80–88%), while in a limited extend excretion occurs through urines (about 13%). Interactions exist for azithromycin and erythromycin ritonavir and saquinavir darunavir/ritonavir cimetidine, fluvoxamine grapefruit bosentan.

Oral intake through the gastrointestinal tract, via tablets, is the frequent administration route and formulation technique, respectively. Although this strategy features advantages such as patient compliance, stability, accurate dosing and easy manufacturing, it also associates with imperfect PK properties i.e., slow dissolution rate and alarming levels of first pass metabolism effect. In order to ameliorate the latter, scientific attention has recently focused on novel oro-dispersible formulations such as tablets, films and chewables. These appear to combine all the advantages of the orally administrated tablets, achieving enhanced patient compliance for the elderly and pediatric populations incorporating fast disintegration/dissolution rates fostering high bioavailability thresholds. In Table 3 and Table 4 the advantages/disadvantages of the several formulations of the various documented administration routes and representative examples of marketed sildenafil products are comprehensively summoned and displayed.

## 9. Conclusions

A yet unresolved issue regarding the use of sildenafil is the utilization of pharmaceutical formulation science in the optimization of the biopharmaceutical profile with the simultaneous amelioration of toxicity and adverse effects. Therefore, the utilization of an optimum drug delivery formulation, aiming to provide effective concentrations selectively to the tissues of interest, is reasonably of interest as the field’s “holy grail”. In order to advance the realization of such controlled administration strategies, encompassing the wide patient satisfaction, research and development efforts will be challenged to adopt a more mechanistic first–principle approach i.e., linking the dots between the PK/PD bioprocess events and the physicochemical particularities.

Expanding on the former statement, the desired vasodilatation response comes after systemic exposure to sildenafil and is enforced on the smooth muscle fibers within the vascular wall. This condition emulates a type of constricted biodistribution model whereas the limiting factors are oriented by the endogenous/imposed blood supply barriers between the central and peripheral vascular system, which in turn limit the cavernous corpus drug transfer. This opinion is in line with the current, phenotypic medical consensus that erectile dysfunction may be a biomarker of atherosclerosis and heart disease.

Moreover, due to the conformational change in PDE5 modulation affinity, the physiological depletion of sildenafil inhibitory action is spatiotemporally impacted. The sildenafil concentration entering the cavernous corpus bound to the PDE receptors could thus remain active, in a counterintuitive fashion to the fraction of the drug retained by plasma albumins. Thus, the binding affinity of sildenafil to PDE-5 rich tissues confers the inhibition of cGMP-activated PDE5 to meet the NO/cGMP requirement. The parameters that favor the attenuated residence of sildenafil in the active site, will be critical for designing novel more selective treatment strategies with reduced side effects in the future.

However, adding to the intrinsic abstruseness that the dynamic ampholytic behavior of sildenafil poses to the pharmacotechnical handling of the API, the development of straightforward formulations methods suitable for the task are not foreseen to be found or might even cannot exist. On the contrary, since the majority of drug interactions with sildenafil are of CYP3A4 origin, a further minimization attempt or even complete evasion of the first pass effect would reduce the unnecessary hepatic metabolic burden. These, non-low hanging fruit yet feasible approaches, if successful, would deliver sildenafil formulations of improved safety and efficacy for the totality of users and in particular for those special populations in need, i.e., the elderly, patients with hepatic or renal impairment and newborn/young subjects suffering from pulmonary hypertension. In essence, sildenafil should be available entailing several administration options, both with local and systemic delivery systems matching each patient’s specific needs.

Regarding future trends, Viagra^®^ 50 mg was the first among erectile dysfunction medications to switch from the prescription-only status, to available over the counter (after specialist consultation) in UK pharmacies since the spring of 2018, under the brand name Connect^®^. This regulatory propensity is certainly expected to continue with more countries entering the reclassification program like New Zealand did with Silvasta^®^. One of the key supporting driving forces beyond the proven safety and democratization of access by affordable pricing, is that sildenafil has become one of the most commonly counterfeited drugs. A simple internet online Google search will yield approximately 37.5 million results offering illegal, unregulated Viagra similars or relevant supplements of at least questionable safety and efficacy for purchase.

Two decades post discovery, sildenafil has already revolutionized the treatment options for pulmonary hypertension and impotency, nourished a vexing perception of sexual performance on demand and had a profound social impact. with the advent of the 4th industrial revolution characterized by the unprecedented singularity of mankind with automation and the competitiveness-fueled, civilization version forged by the virtual social networks, a vast expansion of the sector encompassing the development of novel sildenafil products, may be evidently predicted.

## Figures and Tables

**Table 1:** Indicative references to salts, cocrystals and solvates of sildenafil.

Pharmaceutical Compositions		Ref.
**sildenafil hydrochloride** **sildenafil hydrogensulphate** **sildenafil hemisulphate** **sildenafil hemitratrate** **sildenafil esylate** **sildenafil fumarate**	Salts	[17]
**sildenafil-ACN_I_** **sildenafil ACN_II_**	Solvates	[15]
**sildenafil lactate**		[18,19]
**sildenafil saccharinate CH_3_CN** **sildenafil oxalate** **sildenafil fumarate trihydrate** **sildenafil succinate** **sildenafil glutarate** **sildenafil adipic acid** **sildenafil pimelic acid** **sildenafil suberic acid** **sildenafil sebacic acid**	Solvate Salt Salt Salt Salt Cocrystal Cocrystal Cocrystal Cocrystal	[20]
**Sildenafil-acetylsalicylic-acid**	Cocrystal	[21]
**sildenafil-salicylic acid**	Cocrystal	[22]
**sildenafil resorcinol**	Cocrystal	[23]
**sildenafil Sacharinate with:** **CH_3_NO_2_, CH_3_CN, HCONH_2_, HOC_2_H_4_OH, C_4_H_8_O_2_**	Solvates of salts	[24]
**sildenafil-quercetin** **sildenafil-3,4-dihydroxybenzoic acid** **sildenafil-resorcinol** **sildenafil-tartaric acid** **sildenafil-caffeic acid** **sildenafil-methyl gallate** **sildenafil-3-hydroxybenzoic acid**	Cocrystals	[25]

**Table 2:** Comprehensive collection of advantages and disadvantages of various studied formulations.

Tablets	-Stability -Accurate Dosing -Easy Manufacturing -Small Packaging Size -Easy Handling	-Poor Patient Compliance Due to Swallowing Difficulties -First Pass Metabolism
**Orally disintegrating tablet** **/film**	-disintegration friendly, in the oral cavity, limited volume of saliva -no need of water -easy self-administration	-bitter taste
**Sublingual tablets**	- first past metabolism elimination	
**Chewable tablets**	-easy swallowing -easy self-administration	-first pass metabolism
**Dry foam tablets**	- bioavailability enhancement	-poor patient compliance due to swallowing difficulties -first pass metabolism
**Intranasal**	- first pass metabolism elimination	-poor permeability across nasal mucosa -mucociliary clearance
**Inhalable**	-local action, first pass metabolism elimination	-rapid clearance from the pulmonary compartment
**Transdermal**	-controlled released - first pass metabolism elimination -easy self-administration -avoidance of efflux transporters -improved patient compliance -avoidance of gastrointestinal harsh environment - reduction of dosing frequency -stable plasma levels -extended duration of action	slow absorption rate
**Intravenous**	-highest bioavailability and C_max_	-difficult, demanding process - needle may be painful being the source of infectious disease

**Table 3:** Indicative marketed formulations of sildenafil citrate and diverse administration routes.

Formulation	Route	Dose (mg), (mg/mL)	Excipients	Condition	Name	Market Holder	Authorization Date	EPAR
**Film-coated tablets**	oral	25, 50, 100	Microcrystalline cellulose, Silica, hydrophobic colloidal Croscarmellose sodium, Magnesium stearate Indigo carmine aluminum lake (E132), Sucralose Mannitol, Crospovidone, Polyvinyl acetate, Povidone Flavoring contains: Maltodextrin, Dextrin Natural flavoring contains: Maltodextrin, Glycerol (E422), Propylene glycol (E1520), Lemon flavoring contains: Maltodextrin, Alpha-tocopherol (E307)	ED	Viagra	Pfizer	1998	[127]
**Film-coated tablets**	oral	25, 50, 100	Tablet core Microcrystalline cellulose, Calcium hydrogen phosphate Croscarmellose sodium, Magnesium stearate Film-coat, Poly(vinyl alcohol), Titanium dioxide (E171) Macrogol 3350, Talc	ED	Sildenafil Teva	Teva	2009	[128]
**Orodispersible tablets**	oral	25, 50, 100	Hydroxypropyl cellulose (E463), Mannitol (E421) Aspartame (E951), Neohesperidin-dihydrochalcone (E959), Spearmint oil, Peppermint oil (containing sorbitol (E420)), Crospovidone, Calcium silicate, Magnesium stearate (E572)	ED	Vizarsin	Krka d.d.	2009	[129]
**Orodispersible film**	oral	25, 50, 75, 100	Maltodextrin, Glycerol, Polysorbate 20, Propylene glycol monocaprylate, Polyvinyl acetate dispersion 30%, Lemon and Grapefruit flavors (Lemon essential oil, Citral, Linalool, Grapefruit essential oil, Orange essential oil, Nootkaton, Butylated hydroxyanisol E320, Ascorbic acid E300, Maltodextrin, Arabic gum E414), Sucralose, Titanium dioxide, Indigotine	ED	Sildenafil Sandoz ODF	IBSA	2013	[130]
**Chewable** **tablets**	oral	25, 50, 100	Polacrilin potassium, silica colloidal anhydrous, lactose monohydrate, povidone K-30, aspartame (E951), croscarmellose sodium, peppermint flavor, magnesium stearate, potassium hydroxide (for pH adjustment) or hydrochloric acid (for pH adjustment).	ED	Sildenafil Portfarma	Portfarma	2012	[131]
**Solution for** **injection**	IV	0.8 mg/mL	Glucose Water for injections	PAH	Revatio	Upjohn EESV	2005	[132]
**Powder for** **suspension** **(after** **reconstitution)**	oral	10 mg/mL	Powder for oral suspension: Sorbitol Citric acid anhydrous, Sucralose, Sodium citrate, Xanthan gum, Titanium dioxide (E171), Sodium benzoate (E211), Silica, colloidal anhydrous Grape flavor: Maltodextrin, Grape juice concentrate, Gum acacia, Pineapple juice concentrate, Citric acid anhydrous, Natural flavoring	PAH	Revatio	Upjohn EESV	2005	[132]

**Table 4:** Reverse phase chromatography and operational conditions.

Stationary Phase	Mobile Phase	Flow Rate/Temp	LOD (ng mL^−1^)	UV (nm)	Ref
**Monolithic column Chromolith^®^** **RP-18e, (100 × 4.6 mm I.D)**	Acetonitrile/water, 60/40, *v*/*v*	2 mL min^−1^/Ambient	25	292	[152]
**RP C18**	Phosphate buffer 10 mM (pH 6.5)/MeOH, gradient	1 mL min^−1^/40 °C	1.49	286	[154]
**Bondapak C_18_** **(300 × 3.9 mm, 10 μm)**	CH_3_COONH_4_ 0.2 M (pH 7.0)/CH_3_CN, 50/50 *v*/*v*	1 mL min^−1^/Ambient	0.413	240	[153]
**Spherisorb^®^ silica-C_18_** **(250 × 4.6 mm, 5 μm)**	TEA 0.2% *v/v* (pH = 3) with OPA and ACN (60/40 *v*/*v*)	1 mL min^−1^/Ambient	0.3	230	[155]
**Inertsil C_18_** **(150 × 4.6 mm, 5 μm)**	CH_3_CN/phosphate buffer (70/30 *v*/*v*, pH 7.0)	0.8 mL min^−1^_/_Ambient	1.8	228	[156]
**Inertsil^®^ODS-3;** **(250 × 4.6 mm, 3 μm)**	CH_3_COONH_4_ 0.2M (pH 7.0)/CH_3_CN, 50/50 *v*/*v*	1.0 mL min^−1^/25 °C	3.82	245	[157]
**Poroshell 120 EC-C_18_** **(150 × 4.6 mm, 4 μm)**	CH_3_COONH_4_ 0.03M/CH_3_CN gradient	1.0 mL min^−1^/40 °C	0.67	230	[158]

**Table 5:** Overview of recent bioanalytical methods of sildenafil.

Analyte LC-MS	Sample	Sample Preparation	Analytical Parameters	Detection	LOD/LOQ	Ref
**Sildenafil, *N*-desmethyl sildenafil**	Human plasma	Protein precipitation using acetonitrile	Gradient elution using water and acetonitrile both containing 0.1% *v/v* formic acid Column: Gemini NX-C_18_ (50 × 4.6 mm i.d., 5 μm) + Gemini C_18_ guard column (4 × 3 mm i.d., 5 μm) Flow rate: 0.3 mL min^−1^ Temperature: 35 °C	MS/MS (MRM ^1^)	NM ^2^/2 ng mL^−1^	[164]
**Sildenafil, tadalafil, bosentan, ambrisentan, macitentan**	Human plasma	(1) Protein precipitation using acetonitrile (2) SPE ^3^ (Oasis^®^ HLB 96 well-plate)	Isocratic elution: 5 mM CH_3_COONH_4_/acetonitrile, 50/50 *v*/*v* Column: Symmetry C_18_ (150 × 2.1 mm i.d., 5 μm) Flow rate: 0.3 mL min^−1^ Temperature: 40 °C	MS	NM/1 ng mL^−1^	[165]
**Sildenafil, tadalafil, avanafil, vardenafil**	Human plasma, urine	Magnetic SPE using citric acid coated iron oxide nanoparticles	Gradient elution using 10 mM HCOONH_4_ (pH 4.6) and acetonitrile containing 0.1% formic acid Column: Agilent Poroshell 120 EC-C_18_ (150 × 3.0 mm i.d., 2.7 μm) Flow rate: 0.55 mL min^−1^ Temperature: NM	QTOF-MS/MS	0.74/2.45 ng g^−1^	[166,167]
**Sildenafil, tadalafil, vardenafil and avanafil**	Human plasma, urine	Magnetic SPE using citric acid coated iron oxide nanoparticles	Gradient elution using 10 mM HCOONH_4_ (pH 4.6) and acetonitrile containing 0.1% formic acid Column: Agilent Poroshell 120 EC-C_18_ (150 × 3.0 mm i.d., 2.7 μm) + Agilent Zorbax Eclipse guard column (12.5 × 2.1 mm, 5 μm) Flow rate: 0.55 mL min^−1^ Temperature: NM	QTOF-MS/MS	0.14/0.47 ng g^−1^	[168]
**Sildenafil, tadalafil, vardenafil and avanafil**	Human urine, simulated gastric fluid	Dilution	Gradient elution using 10 mM HCOONH_4_ (pH 4.6) and acetonitrile containing 0.1% formic acid Column: Agilent Poroshell 120 EC-C_18_ (150 × 3.0 mm i.d., 2.7 μm) + Agilent Zorbax Eclipse guard column (12.5 × 2.1 mm, 5 μm) Flow rate: 0.55 mL min^−1^ Temperature: 40 °C	QTOF-MS/MS	2.19/7.28 ng g^−1^	[170,171,172,173]
**Sildenafil, rosiglitazone**	Rat plasma	Protein precipitation using methanol	Gradient elution using water and methanol both containing 0.1% formic acid Column: Kinetex C_18_ (50 × 2.1 mm i.d., 1.3 μm) Flow rate: 0.25 mL min^−1^ Temperature: 40 °C	MS/MS (MRM)	NM/5 ng mL^−1^	[174,175,176]
**Sildenafil, *N*-desmethyl sildenafil**	Human plasma	Protein precipitation using acetonitrile	Gradient elution using water and acetonitrile both containing 0.05% formic acid Column: Acquity UPLC^®^ HSS T3 C_18_ (150 × 2.1 mm i.d., 1.8 μm) Flow rate: 0.4 mL min^−1^ Temperature: 40 °C	MS/MS (MRM)	1.95/3.9 ng mL	[30]
**Sildenafil, mirodenafil, tadalafil, udenafil, vardenafil and their metabolites**	Animal hair	Digestion with 5M HCl methanolic solution followed by mixed-mode SPE using C_18_ and strong ion exchange polymeric sorbents	Gradient elution using water and acetonitrile containing 0.1% formic acid Column: Agilent Poroshell 120 EC-C_18_ (50 × 3.0 mm i.d., 2.7 μm) Flow rate: 0.3 mL min^−1^ Temperature: 30 °C	MS/MS (MRM)	0.05/0.1 ng mg^−1^	[177,178,179]
**Sosentan, ambrisentan,** **sildenafil, tadalafil**	Human plasma	SPE using Oasis WAX cartridges	Isocratic elution using 5 mM CH_3_COONH_4_ (pH 5.0)/acetonitrile, 55/45 *v*/*v* Column: Cadenza CD-C_18_ (75 × 2.0 mm i.d., 3 μm) + Phenomenex Security guard column Flow rate: 0.2 mL min^−1^ Temperature: 40 °C	MS/MS (MRM)	NM/2 ng mL^−1^	[180]
**Sildenafil**	Human plasma	Protein precipitation using acetonitrile followed by heating at 60 °C	Gradient elution using 50 mM CH_3_COONH_4_/3% trifluoroacetic acid/methanol/acetonitrile, 68/2/15/15 *v*/*v*/*v*/*v* Column: Agilent Poroshell 120 EC-C_18_ (50 × 3 mm i.d., 2.7 μm) Flow rate: 0.3 mL min^−1^ Temperature: 30 °C	MS/MS (MRM)	7.25/10 ng mL^−1^	[181]
**Sildenafil**	Dried blood spot	LLE ^4^ using diethyl ether	Isocratic elution using 2 mM CH_3_COONH_4_ (pH 5.0)/acetonitrile, 65/35 *v/v* Column: BEH C_18_ (50 × 2.1 mm i.d., 1.7 μm) Flow rate: 0.3 mL min^−1^ Temperature: 40 °C	MS/MS (MRM)	NM/5 ng mL^−1^	[182]
**Sildenafil, *N*-desmethyl sildenafil**	Human plasma	LLE using methyl terb-butyl ether	Isocratic elution using 0.02% formic acid/acetonitrile, 30/70 *v/v* Column: Thermo Hypersil Gold (50 × 2.1 mm i.d., 5 μm) Flow rate: 0.5 mL min^−1^ Temperature: 35 °C	MS/MS (MRM)	NM	[183]
**Sildenafil**	Human plasma	SPE using Sep-Pak tC18	Isocratic elution using 5 mM ammonium formate/acetonitrile, 60/40 *v/v* Column: Thermo Hypersil Gold (50 × 2.1 mm i.d., 5 μm) Flow rate: 0.5 mL min^−1^ Temperature: 35 °C	MS	NM/5 ng mL^−1^	[184]
***HPLC-UV***						
**Sildenafil, avanafil, apomorphine, trazodone, yohimbine, tramadol, dapoxetine**	Human plasma	Protein precipitation using acetonitrile	Gradient elution using sodium octanesulfonate, EDTA aqueous solution (pH 3.0) and acetonitrile or ethanol Column I: Chromolith Performance RP-18e (100 × 4.6 mm i.d. Column II: Poroshell core-shell EC-C_18_ (150 × 4.6 mm i.d., 2.7 μm) Flow rate: 1 or 2 mL min^−1^ Temperature: 35 °C	UV@210nm	200/500 ng mL^−1^ (using Column I) 200/500 ng mL^−1^ (using Column II)	[167]
**Sildenafil, tramadol**	Rabbit plasma	SPE using Oasis HLB	Isocratic elution using 10 mM phosphate buffer (pH 7.5)/acetonitrile, 55/45 *v*/*v* Column: ODS Discovery HS C_18_ (150 × 4.6 mm i.d. 5 μm) Flow rate: 0.8 mL min^−1^ Temperature: Ambient	UV@220nm	0.01/0.03 μg mL^−1^	[169]
**Sildenafil**	Rat plasma	Protein precipitation	Isocratic elution using a mixture of acetonitrile and water (57.5/42.5 *v*/*v*) containing 0.675 mL trimethylamine (pH 7 with H_3_PO_4_) Column: Sepax Gp-C_18_ (150 × 4.6 mm i.d. 5 μm) Flow rate: 1 mL min^−1^ Temperature: 30 °C	UV@230nm	NM/20 ng mL^−1^	[171]
**Sildenafil, *N*-desmethyl sildenafil**	Human plasma	LLE using ethyl acetate	Isocratic elution using a mixture of 30 mM phosphate buffer (pH 6.0)/acetonitrile, 53/47 *v*/*v* Column: Inertsil ODS2 C_18_ (150 × 4.6 mm i.d. 5 μm) Flow rate: 1 mL min^−1^ Temperature: 25 °C	UV@230nm	0.5/1 ng mL^−1^	[172]
**Sildenafil**	Human plasma	LLE using diethylacetate followed by back-extraction with 5% HClO_4_ aqueous solution	Isocratic elution using a mixture of water and acetonitrile (63/37 *v*/*v*) containing 0.1% TEA (pH 7.7) Column: Hypersil BDS-C_18_ (150 × 4.6 mm i.d. 5 μm) Flow rate: 1 mL min^−1^ Temperature: 25 °C	UV@230nm	NM/2 ng mL^−1^	[175]
**Sildenafil**	Rat plasma	LLE using ethyl acetate/hexane (30/70 *v*/*v*) followed by back-extraction with mixture of methanol/0.1 M H_2_SO_4_ aqueous solution (10/90 *v*/*v*)	Isocratic elution using a mixture of 50 mM KH_2_PO_4_ (pH 4.5) and acetonitrile, 75/25 *v/v* Column: Supelcosil PCN cyanopropyl (250 × 4.6 mm i.d. 5 μm) Flow rate: 1 mL min^−1^ Temperature: 22 °C	UV@230nm	5/10 ng mL^−1^	[176]
**aliskiren, prasugrel, rivaroxaban, rednisolone, propranolol, ketoprofen, nifedipine, naproxen, terbinafine, ibuprofen, diclofenac, sildenafil, acenocoumarol**	Human urine	SPE using 17 different silica- and polymeric-based sorbents	Gradient elution using water and acetonitrile both containing 0.05% trifluoroacetic acid Column: Hypersil Gold C_18_ (50 × 2.1 mm i.d., 1.9 μm) Flow rate: 0.5–1.0 mL min^−1^ Temperature: 25 °C	UV@228nm	66/198 ng mL^−1^	[179]
**Sildenafil, *N*-desmethyl sildenafil**	Human plasma	LLE using ethyl acetate	Isocratic elution using a mixture of 30 mM KH_2_PO_4_ (pH 4.5) and acetonitrile, 53/47 *v*/*v* Column: μBondapack C18 (150 × 3.9 mm i.d. 5 μm) Flow rate: 0.8 mL min^−1^ Temperature: 21 °C	UV@230nm	1/10 ng mL^−1^	[185]
**Sildenafil, vardenafil, aildenafil**	Human plasma	Ionic liquid-based dispersive liquid liquid microextraction followed by back-extraction with 10% acetic acid	Isocratic elution using a mixture of water and methanol both containing 1% acetic acid, 60/40 *v/v* Column: Shimazdzu RP-C_18_ (250 × 4.6 mm i.d. 5 μm) Flow rate: 1.2 mL min^−1^ Temperature: NM	UV@254nm	0.17/NM	[186]
***Voltammetry***						
**Sildenafil**	Human serum	Dilution	Screen-printed electrode with carbon working and auxiliary electrodes and silver reference electrode Potential range: −0.75 to 1.55 V Scan rate: 175 mV s^−1^ Sample medium: 0.15 M acetate buffer (pH 5.0)	−	5.9 × 10^−10^/2.0 × 10^−9^ mol L^−1^	[151]
**Sildenafil**	Human serum	NM	Square wave voltammetry using polycrystalline gold (surface area 0.5 cm^2^), gold wire and saturated calomel electrode as working, counter and reference electrodes respectively Sample medium: 0.05 M NaHCO_3_	−	0.031/0.106 μmol L^−1^	[173]
**Sildenafil**	Simulated human urine	Dilution	Cyclic voltammetry using screen-printed glassy carbon electrode modified gold nanoparticles via electrodeposition Sample medium: Britton-Robinson buffer (pH 7.3)	−	5.2 × 10^−10^ mol L^−1^/NM	[178]
***Batch Spectrophotometry***						
**Sildenafil**	Human urine	Dispersive solid-phase microextraction using Mn@ CuS/ZnS nanocomposite loaded on activated carbon	−	NM	2.5/8.35 ng mL^−1^	[181]

^1^ MRM: multiple reaction monitoring. ^2^ NM: not mentioned. ^3^ SPE: solid phase extraction. ^4^ LLE: liquid liquid extraction

## Data Availability

Not applicable.
